# Asymmetric chromatin accessibility underlies anterior-posterior axis specification in moth fly embryos

**DOI:** 10.1371/journal.pbio.3003896

**Published:** 2026-08-06

**Authors:** Ezra E. Amiri, Muzi Li, Ayse Tenger-Trolander, Maxwell Devine, Alexander T. Julian, Koray Kasan, Sheri A. Sanders, Shelby A. Blythe, Urs Schmidt-Ott

**Affiliations:** 1 Department of Organismal Biology and Anatomy, The University of Chicago, Chicago, Illinois, United States of America; 2 Department of Biology, Illinois Institute of Technology, Chicago, Illinois, United States of America; 3 University of Notre Dame, Department of Biological Sciences, Notre Dame, Indiana, United States of America; 4 Kallista Consulting, Niles, Michigan, United States of America; 5 Department of Molecular Biosciences, Northwestern University, Evanston, Illinois, United States of America; 6 National Institute for Theory and Mathematics in Biology, Northwestern University and The University of Chicago, Chicago, Illinois, United States of America; Institut Pasteur, FRANCE

## Abstract

Establishing the anterior-posterior (AP) body axis is a fundamental process during embryogenesis, and the fruit fly, *Drosophila melanogaster*, provides one of the best-known case studies. But for unknown reasons, different species of flies (Diptera) establish the AP axis through unrelated, structurally distinct anterior determinants. The anterior determinant of Drosophila, Bicoid (Bcd), initiates symmetry-breaking during nuclear cleavage cycles (NCs) when ubiquitous pioneer factors, such as Zelda (Zld), drive zygotic genome activation (ZGA) at the level of chromatin accessibility by nucleosome depletion. While Bcd engages in a concentration-dependent competition with nucleosomes at the loci of a small set of transcription factor (TF) genes that are expressed in the anterior embryo, it remains unknown whether unrelated anterior determinants of other fly species function in the same way and target homologous genes. We have examined the symmetry-breaking mechanism of a moth fly, *Clogmia albipunctata*, in which a maternally expressed transcript isoform of the pair-rule segmentation gene *odd-paired* serves as an anterior determinant. We provide a de novo assembly and annotation of the Clogmia genome, report changes in chromatin accessibility during the nuclear cleavage cycles (NCs) of consecutive blastoderm stages, and describe how Clogmia’s orthologs of *zelda* (*Cal-zld*) and *odd-paired* (*Cal-opa*) affect chromatin accessibility and gene expression. We document extensive opening and closing of chromatin regions during cleavage cycles of blastoderm, important roles of *Cal-zld* in opening chromatin and driving zygotic gene expression, and show that maternal *Cal-opa* activity initiates zygotic symmetry-breaking along the AP axis by driving chromatin accessibility and expression at Clogmia’s *homeobrain* and *sloppy-paired* loci. These genes are not known as key targets of Bcd but may serve a more widely conserved role in the initiation of anterior pattern formation, given their early anterior expression and function in head development in insects.

## Abstract 2

This study establishes resources for dissecting developmental gene networks in a non-traditional dipteran (fly) model organism and reveals both similarities and significant differences in the fundamental developmental processes of early embryogenesis including zygotic genome activation and primary axis specification.

## Introduction

The insect order of flies (Diptera) offers excellent opportunities for comparative study of developmental mechanisms, as many dipteran species can be reared cost-effectively and because the fruit fly, *Drosophila melanogaster*, provides a well-established model for functional comparisons at the molecular and cellular level. Dipteran embryos are morphologically similar across the clade [1,2]. However, the mechanisms of axis specification [3,4], primordial germ cell specification [3], extraembryonic tissue specification [5,6], morphogenesis [5,7–11], and sex determination [12–15] differ between dipteran species. Explaining such unexpected plasticity in developmental gene networks requires functional comparisons, which can reveal underlying general principles and mechanisms of evolutionary change. Anterior-posterior (AP) axis specification, a long-standing model for pattern formation and gene regulation in Drosophila [16–24], is particularly suitable for comparative functional studies because the syncytial nature of early dipteran embryos facilitates visualization and perturbation of gene activity in nontraditional model organisms. Many dipterans establish the embryo’s head-to-tail polarity through transcription factors (TFs) that diffuse from maternally localized mRNA and form AP concentration gradients that regulate gene expression in a dose-dependent manner [25,26]. These anterior determinants prevent the formation of bicaudal embryos that lack head and thorax (double abdomen). Irrespective of this fundamental role, unrelated anterior determinants have been found in different fly species. While the anterior determinant of Drosophila and many other brachyceran fly species is encoded by a homeobox gene, *bicoid* [27–32], diverse zinc finger genes take on this role in lower dipterans, including *pangolin* (dipteran ortholog *Tcf* gene family) in anopheline mosquitoes and crane flies, *cucoid* in culicine mosquitoes, *panish* in certain harlequin flies (Chironomini), and *odd-paired* (dipteran ortholog of *Zic* gene family) in moth flies (Psychodidae) [3,4]. The phylogenetic occurrence of these anterior determinants indicates that they have been frequently replaced during the dipteran radiation. Moreover, the anterior determinant proteins identified so far differ widely in their structure and DNA-binding domains. Bicoid (Bcd) binds DNA via its homeodomain, Pangolin (Pan) through HMG and C-clamp domains, Panish through a C-clamp domain, and Odd-paired (Opa) and Cucoid through distinct C2H2 zinc finger domains [33–38]. This molecular diversity prompted us to ask whether the mechanisms of dipteran anterior determinants and their target genes differ between species or whether they are conserved due to contextual or downstream network constraints. In this study, we address these questions by analyzing the mechanism of action of the anterior determinant of the moth fly *Clogmia albipunctata*, a C2H2 zinc finger protein encoded by the *zic* gene family member *odd-paired* [3] and comparing it to the anterior determinant of *Drosophila melanogaster*, a homeodomain protein encoded by *bicoid* [27], which is missing in Clogmia and other midges.

Fly embryos begin embryonic development with a series of synchronized intravitelline nuclear divisions. The nuclei are then arranged in a monolayer between the egg membrane and the central yolk sac. This ‘syncytial blastoderm’ undergoes additional nuclear cleavage cycles before it cellularizes and initiates gastrulation. The embryos of Drosophila and Clogmia undergo 13 nuclear division cycles, form the syncytial blastoderm at the end of nuclear cycle (NC) 9 (Clogmia) or the beginning of NC10 (Drosophila), and cellularize it during NC14, thereby ending the syncytial phase of embryonic development and setting the stage for gastrulation.

In Drosophila, only a few hundred genes are expressed before the midblastula transition in NC14 [39–41], when gap phases are added to the now desynchronized mitotic cycle and the embryo transitions from maternal to zygotic control of development [42]. Genes expressed during NC8-NC13 include regulators of the cell cycle, sex determination, dosage compensation, and early pattern formation [43–45]. However, widespread zygotic genome activation (ZGA) is delayed until NC14 when the prolongation of the mitotic cycle enables the expression of longer transcripts and provides more time for the regulation of global changes in chromatin accessibility [45–47]. After each DNA replication cycle, nucleosome formation reduces chromatin accessibility, creating a barrier to TF binding. This barrier is overcome by a special class of sequence-specific TFs capable of binding nucleosome occupied DNA and destabilizing nucleosomes [48,49]. The major driver of chromatin accessibility during Drosophila’s nuclear cleavage cycles is Zelda (Zld), a ubiquitously expressed zinc finger protein [44,50–53]. Zld’s activity is required throughout ZGA and enables the binding of other TFs, including those involved in embryonic patterning [51,54–58]. Two additional pioneer TFs, GAGA Factor (GAF) and CLAMP, act independently and cooperatively with Zld to establish open chromatin and drive zygotic gene expression once major ZGA is underway [59–62].

At the beginning of the blastoderm stage, chromatin accessibility measured by ATAC-seq (assay for transposase-accessible chromatin with sequencing) is largely homogeneous across the embryo. Indeed, Zld establishes chromatin accessibility ubiquitoulsy in the embryo at many sites that are targeted by Bcd [55,57,63–69]. However, Bcd’s role in AP patterning becomes indispensable after blastoderm formation at the beginning of NC10 [70], and a small cohort of early segmentation gene enhancers that drive Bcd-dependent anterior expression exhibits anteriorly restricted accessibility [35,71–77]. The underlying mechanism has been inferred by measuring accessibility and activity of the Bcd-dependent proximal P2 enhancer element of *hunchback*, an early target of Bcd, under experimentally modulated Bcd input and nucleosome stability at the enhancer [78]. Bcd engages in a concentration-dependent competition with nucleosomes to ‘open’ otherwise inaccessible regulatory regions. Bcd activity is thereby effectively breaking symmetry along the AP axis at the level of chromatin accessibility, well before the bulk of ZGA and pattern formation occurs [79]. However, Bcd remains active during the major wave of ZGA, when the embryo establishes a segmented body plan through a regulatory network of TF encoding genes, known as gap and pair-rule genes [20,22,80,81].

In Clogmia, the cleavage cycles prior to NC14 are ~3 times slower than in Drosophila [82,83] but its segmented body plan appears, like in Drosophila, during NC14 [83,84]. However, the activity window of its anterior determinant, a maternal transcript isoform of Clogmia’s *odd-paired* gene *Cal-opa*, is constrained by its zygotic function in the pair-rule gene network, which appears to be conserved [3,85]. Knockdown of the maternal *Cal-opa* transcript by isoform-specific RNA interference (RNAi) results in double abdomens that are normally segmented. Conversely, ectopic posterior expression of *Cal-opa* by mRNA injection can induce a bicephalic (double head) phenotype, but this phenotype is only observed when embryos are injected prior to blastoderm formation; delayed injection of the same mRNA during the syncytial blastoderm stage (4 hours after egg activation) fails to induce ectopic head structures [3]. Therefore, early Cal-Opa activity is both necessary and sufficient for establishing head-to-tail polarity of the Clogmia embryo and does not overlap with the function of the zygotic Cal-opa transcript isoform, which is expressed at NC14 and encodes a nearly identical protein.

Since zygotic Opa pioneers chromatin accessibility in late NC14 Drosophila embryos to advance the progression of segmentation and dorsoventral patterning [76,86], we asked whether the maternal *Cal-opa* gradient of Clogmia breaks axial symmetry by driving chromatin accessibility at gene loci that are transcribed in the anterior blastoderm before the beginning of NC14 and whether these genes are homologous to Bcd target genes. To set the stage for addressing these questions, we first assembled and annotated the Clogmia genome and identified homologs of Drosophila’s segmentation genes. We then characterized chromatin accessibility in single wild-type embryos at NC11, NC12, NC13, and both early and late NC14 to predict putative enhancer and promoter regions and to capture their stage-dependent accessibility. These experiments revealed opening and—unlike in Drosophila [79]—a lesser but substantial amount of chromatin closing during the roughly 3 times longer cleavage cycles of the syncytial blastoderm of Clogmia. We then assessed how chromatin accessibility and zygotic transcription are affected in embryos with reduced *Cal-zld* or reduced maternal *Cal-opa* activity. These experiments revealed (in conjunction with motif enrichment analyses) an important, likely direct, role of Cal-Zld in opening chromatin and activating zygotic gene expression in Clogmia, and identified homologs of *sloppy-paired* and *homeobrain* as target genes of maternal Cal-Opa, both in terms of chromatin accessibility and zygotic gene expression. Finally, we show that Clogmia’s *homeobrain* (*Cal-hbn*) and *sloppy-paired* homologs (*Cal-slp1*, *Cal-slp2*, *Cal-slp3*) function in complementary domains across the anterior half of the blastoderm, suggesting that these genes initiate zygotic symmetry-breaking along the AP axis downstream of Clogmia’s anterior determinant.

## Results

### De novo assembly and annotation of the genome of *Clogmia albipunctata* reveals multiple lineage-specific duplications of early segmentation gene homologs

**Fig 1:**
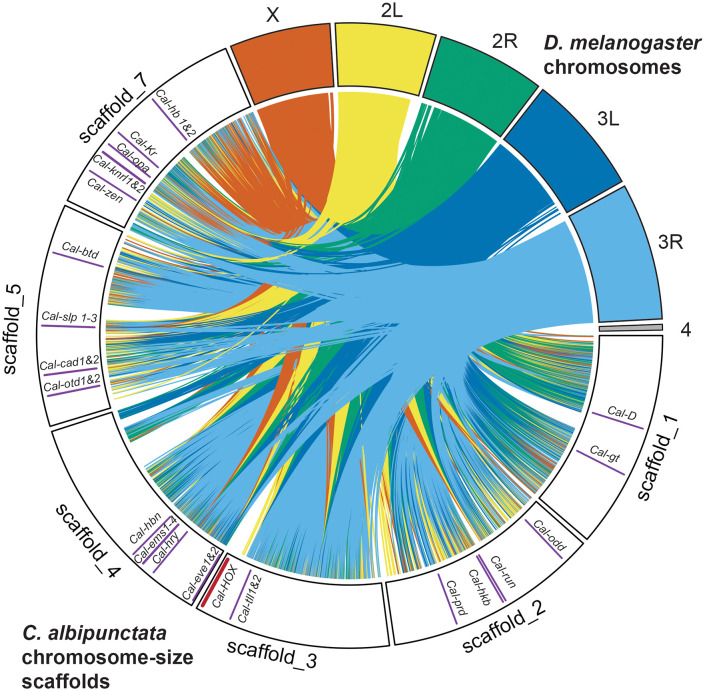
Synteny analysis of *Drosophila melanogaster* chromosomes and *Clogmia albipunctata* scaffolds. Synteny analysis between *Clogmia albipunctata* chromosomes-sized scaffolds *and Drosophila melanogaster* chromosomes. Groups of collinear genes are represented by contact lines connecting the positions shared between *D. melanogaster* chromosomes and *C. albipunctata* scaffolds. Colors correspond to *D. melanogaster* chromosomes. Positions of developmental genes of interest are indicated by solid purple bars. Position of the HOX gene cluster indicated by a solid brown bar.

We generated a high quality de novo genome assembly of *Clogmia albipunctata* with 6 chromosome-size scaffolds (Fig 1, Table 1, S1 Appendix), consistent with the known chromosome number in this species [87,88]. The total length of the chromosome-size scaffolds is 304.3 Mb, close to genome size estimates based on flow cytometry data (316.6 Mb) [89]. An additional 268 small scaffolds (<1 MB) contained mostly repetitive sequences. We annotated this new reference genome using RNA-seq transcripts from embryonic, larval, pupal, and adult stages (S1 Table), protein sequences, and ab initio gene prediction software, and identified 15,047 protein coding genes. The annotated genome recovered 88%−94% of Complete Benchmarking Universal Single-Copy Orthologs (BUSCO) (Table 2), in line with recently published dipteran genomes [90–92]. While synteny between the chromosomes within the Cyclorrhapha clade can be highly conserved (time of divergence ~145 million years ago) [91], synteny between the chromosomes of *D. melanogaster* and *C. albipunctata* (divergence time ~250 million years ago) was essentially lost (Fig 1).

**Table 1:** *Clogmia albipunctata* reference assembly statistics and quality metrics.

Reference Assembly Statistics	
Total length (bp)	320,011,659
Number of scaffolds	274
Scaffold N50 (bp)	51,218,107
Scaffold L50	4
# of Ns per 100kb	0.19
GC (%)	32.34%
Heterozygosity (%)	0.018%–0.038%
Masked (%)	48.55%
Eukaryotic BUSCOs recovered (%)	98.04%

Completeness and continuity of the assembly are assessed by Benchmarking Universal Single-Copy Orthologs (BUSCO) and scaffold N50 and L50, respectively. Eukaryotic BUSCOs recovered: the percentage of complete universal orthologs from the eukaryotic BUSCO dataset identified in the assembly. Scaffold N50: the length (in bp) of the shortest scaffold such that all scaffolds of equal or greater length account for at least 50% of the total assembly length [93]. L50: the minimum number of scaffolds whose collective length accounts for 50%of the total assembly length [94].

**Table 2:** *Clogmia albipunctata* annotation statistics and BUSCO scores.

Annotation Statistics	
Number of coding genes	15,047
Number of mRNAs	31,113
Number of mRNAs with 3′ and 5′ UTR	25,724
Mean mRNAs/gene	2.1
Mean gene length (bp)	9,163
**Complete Single-Copy BUSCOs**	
Eukaryota (*n* = 255)	93.70%
Metazoa (*n* = 954)	93.30%
Insecta (*n* = 1,367)	94.00%
Endopterygota (*n* = 2,124)	92.90%
Diptera (*n* = 3,285)	88.80%

The quality metrics include the percentage of complete universal orthologs identified in the annotation across five BUSCO datasets. The number of genes in each lineage-specific BUSCO database is shown in parentheses.

Next, we interrogated the genome for the presence of predicted early segmentation genes (S2 Appendix). As expected, no *bicoid* ortholog was found but we recovered two putative Hox class 3 genes to which *bicoid* belongs [95], including the previously reported homolog of *zerknüllt* (*Cal-zen*) [96] on scaffold 7 and a more diverged potential *Cal-zen* paralog in position 3 of the Hox gene complex on scaffold 3 (evm.TU.scaffold_3.2943). Our annotation also included homologs of *Drosophila melanogaster*’s early segmentation genes, including gap genes (*tailless*, *huckebein*, *orthodenticle/ocelliless*, *empty spiracles*, *buttonhead*, *hunchback*, *Krüppel*, *giant,* and *knirps*), pair-rule genes (*even-skipped*, *fushi tarazu*, *odd-skipped*, *runt*, *sloppy-paired*, *paired, hairy,* and *odd-paired*), and other important regulators of the segmentation network (*caudal*, *Dichaete*) [20,22]. Some of these genes have more than one copy in the Clogmia genome, including 2 copies of *hunchback* (*Cal-hb1, Cal-hb2*), *knirps/knirps-like* (*Cal-kni/knrl1, Cal-kni/knrl2*), *orthodenticle* (*Cal-otd1, Cal-otd2*), *even-skipped* (*Cal-eve1, Cal-eve2*), *tailless* (*Cal-tll1, Cal-tll2*) and *caudal* (*Cal-cad 1, Cal-cad2*), 3 copies of *sloppy-paired1/2/fd19B* (*Cal-slp1, Cal-slp2,* and *Cal-slp3*), and 4 copies of *empty spiracles* (*Cal-ems1, Cal-ems2, Cal-ems3,* and *Cal-ems4*) (Fig 1, S2 Table)*.* Such gene duplications are a potential source of redundancies in Clogmia’s segmentation gene network (see below).

### Patterns of chromatin opening and closing during nuclear cleavage cycles in Clogmia suggest coordinated regulation of groups of genes

To identify accessible chromatin before and during the main wave of ZGA, we performed single-embryo ATAC-seq at NC11, NC12, NC13, and NC14 (before cellularization), and Late NC14 ~20 min before gastrulation. The embryos were staged in vivo using developmental time after egg activation at 25 °C and morphological criteria as previously described [83] and validated under our laboratory conditions. At least three replicates were performed for NC11 though NC14, and two replicates were performed for Late NC14. We pooled the ATAC-seq data from all stages to create a master peak list of 32,157 open chromatin regions (peaks), using MACS2 [97]. We also computed the number of peaks for each developmental stage excluding stage-specific peaks that were not recovered as significant in the master peak list. In this analysis, we found that the overall number of peaks increases with each NC, suggesting that the chromatin landscape is continuously changing (Table 3, S3 Appendix). Since promoter accessibility does not necessarily indicate gene expression [98], we divided the accessible chromatin regions into transcription start site (TSS) proximal regions (within 500 bp of TSS) and TSS distal regions (>500 bp) to facilitate the distinction of open promoters and other open *cis-*regulatory elements. ATAC-seq peaks that only mapped to intergenic or intronic sequence were treated as candidate enhancers or silencers (henceforth collectively referred to as enhancers). The relative proportion of candidate enhancers increased by about 10% from NC11 (46.8%) to NC14 (58%) and then decreased from NC14 to Late NC14 (52.7%) (S1 Fig). These observations suggest similar dynamic changes in chromatin accessibility during ZGA in Clogmia and Drosophila [99,100].

**Table 3:** Comparison of chromatin accessibility peaks, nuclear cycle length in minutes, and haploid genome size of *Clogmia albipunctata* and *Drosophila melanogaster.*

	*Clogmia albipunctata*				
	NC11	NC12	NC13	NC14	Late NC14
Peaks	5,013	5,926	7,586	16,147	11,628
Duration	30 m	37 m	1 h 13 m	1 h 47 m	
Genome size	~304 Mb				
	*Drosophila melanogaster*				
	NC11	NC12	NC13	NC14	
Peaks	3,084	6,740	9,824	13,226	
Duration	10 m	12 m	21 m	1 h 5 m	
Genome size	~137 Mb				

Peaks for *Drosophila melanogaster* are based on [71,79] and nuclear cycle lengths are based on [83].

**Fig 2:**
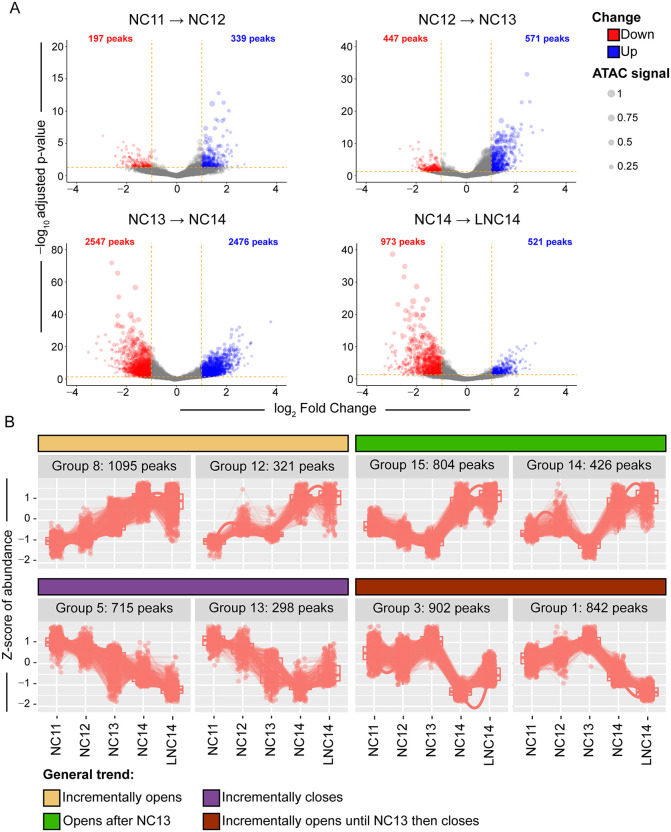
Changes in chromatin accessibility during NC11 to Late NC14. **A)** Volcano plots of differentially accessible ATAC-seq peaks between consecutive developmental stages. Significant change of adjusted *p*-value ≤ 0.05 and |log2 fold change| ≥ 1are highlighted in red (log2 fold change ≤ −1) or blue (log2 fold change ≥ 1). **B)** Dynamic peak groups were identified using DEGreport (Pantano, 2017) (R package version 1.38.5). Y-axis, Z-score of abundance. X-axis, developmental stage. Underlying data is available in S1 Data and NCBI (bioproject: PRJNA1198932).

To identify peaks with dynamic patterns of chromatin accessibility, we compared the degree of accessibility of each peak between successive timepoints (Fig 2A) using DESeq2 [101]. This analysis identified a total of 6,930 dynamically regulated peaks (adjusted *p*-value ≤ 0.05 and |log_2_ fold change| ≥ 1; 21.6% of all 32,157 peaks) and enabled us to quantitatively determine the behavior of each peak (i.e., maintaining, gaining, or losing accessibility) between consecutive developmental stages. We then clustered dynamic peaks into groups based on shared changes in accessibility from stage to stage and delineated 21 dynamic groups (S2 Fig). Nearly 80% (5,403/ 6,930) of the dynamic peaks fell into one of 8 groups, which reveal the major accessibility trends we observe in the data (Fig 2B). Many of the dynamic peaks closed (14.6%; Groups 5, 13) or opened (20.4%; Groups 8, 12) gradually over time. The remaining dynamic peak groups exhibited more complex dynamics between NC11 and Late NC14, commonly with an inflection at NC13 (e.g., Groups 3, 1, 15, 14; see also S2 Fig). These observations suggest that remodeling of chromatin accessibility (both opening and closing) between NC11 and Late NC14 at individual peaks often follows patterns that are shared by many peaks and may reflect co-regulation of the associated genes.

### *Cal*-*zld* dependent gene expression and chromatin accessibility

Previous studies in Drosophila have shown that temporal changes in chromatin accessibility are regulated by the interplay of TFs that can interact with nucleosome-bound DNA (pioneer factors) and TFs that have limited or no ability to initiate chromatin accessibility [47,54]. The earliest changes in chromatin accessibility are primarily driven by the pioneer factor Zld [44,52,56,99,102]. As ZGA accelerates, additional pioneer factors, such as GAF [61], CLAMP [59,60], and Opa [76,86] are required for maintaining, modulating, or gaining chromatin accessibility. In Clogmia, a motif enrichment analysis of peaks with the MEME software package [103,104] revealed an enrichment of a Zelda-binding motif (5′-CAGGTA), GA-rich motifs recognized by GAF and CLAMP [61,105–107], in addition to (CA)_n_ motifs recognized by Combgap (Cg) [108], and others (S3 Fig). Opa-binding motifs were not recovered in this analysis. These results are comparable to findings in Drosophila [76,79,86] and suggest that motif enrichment within chromatin accessible at ZGA is very similar between Drosophila and Clogmia.

In early Drosophila embryos, loss of Zld causes widespread reduction of chromatin accessibility and failure to activate a large cohort of early zygotic genes, including genes required for pattern formation and cellularization [44,52]. Both motif affinity and occupancy positively correlate with Zld’s pioneering activity [44,52,54,109]. Homologs of *zelda* have been identified in many insects and some crustaceans [110], and several transcriptomic studies suggest that Zld’s function as pioneer TF during ZGA is conserved across insects [111–115]. However, these comparative studies did not directly test for Zld-dependent effects on chromatin accessibility. We wanted to know how *zelda* might affect chromatin accessibility in regions that open or close as the Clogmia embryo reaches NC14. We identified a single ortholog of *zelda* in the Clogmia genome (*Cal-zld*; S4 Fig) and observed high levels of *Cal-zld* expression at preblastoderm and blastoderm stages (S5 Fig). To examine phenotypic effects of *Cal-zld* depletion, we injected batches of ~30 embryos with *Cal-zld* dsRNA (double-stranded RNA) within the first hour of development (NC2/3) and monitored their development in vivo until the end of NC14 (roughly 7–7.5 hours after injection). Preblastoderm mortality of injected embryos (33/59; 56%) was higher than in the uninjected controls (6/22; 27%), but this difference may have been caused by unspecific side effects of the injection procedure. Across three independent replicates, a subset of the injected embryos reached NC14 but failed to cellularize and developed abnormally (S1 Movie). This phenotype is comparable to the phenotype of *zld* mutant Drosophila embryos (S2 Movie) [56] and suggests that Zld performs similar developmental roles in both species.

**Fig 3:**
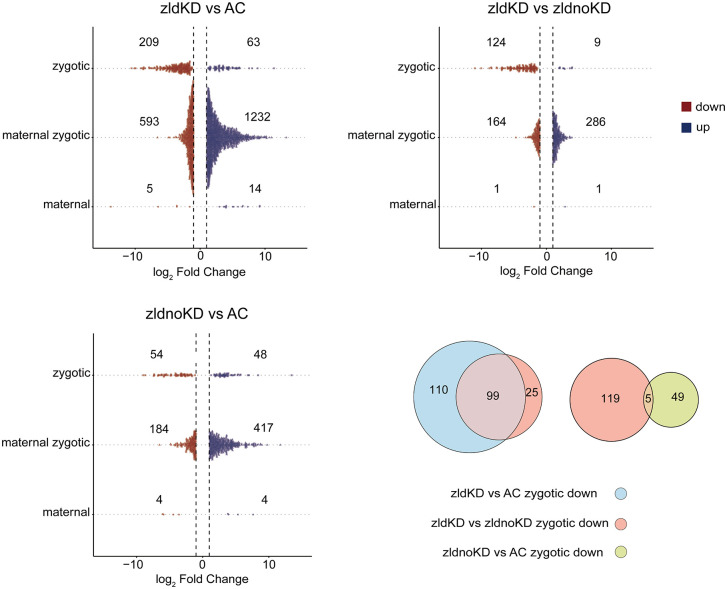
Cal-Zld promotes zygotic transcription. Individual panels show the number of downregulated and upregulated genes when comparing *Cal-zld* RNAi embryos with efficient *Cal-zld* knockdown to alignment controls (zldKD vs. AC) or to *Cal-zld* RNAi embryos without *Cal-zld* knockdown (zldKD vs. zldnoKD). The comparison of *Cal-zld* RNAi embryos without *Cal-zld* knockdown to alignment controls (zldnoKD vs. AC) serves as a negative control. On the y-axis, genes are grouped based on maternal, maternal and zygotic, or zygotic expression (see Materials and methods). Log_2_ fold change is shown on the x-axis. Only genes with an adjusted *p*-value ≤ 0.05 and |log_2_ fold change| ≥ 1 are shown. Venn diagrams show overlap of downregulated zygotic genes between zldKD vs. zldnoKD (pink) and zldKD vs. AC (blue) or zldnoKD vs. AC (green). Underlying data is available in S2 Data and NCBI (bioproject: PRJNA1198932).

To assess *Cal-zld*’s role in shaping chromatin accessibility and zygotic transcription, we adapted our ATAC-seq protocol to isolate RNA from single-embryo ATAC-seq library preparations and used the RNA-seq data to quantify *Cal-zld* expression levels in Late NC14 *Cal-zld* RNAi embryos and uninjected control embryos (S6 Fig and see Materials and methods). We then compared gene expression (RNA-seq data) and chromatin accessibility (ATAC-seq data) between RNAi embryos with knockdown efficiencies greater than 75% (*n* = 4; zldKD group), RNAi embryos in which no knockdown of *Cal-zld* was detected (*n* = 3; zldnoKD group), and uninjected embryos that were otherwise treated like the RNAi embryos (*n* = 5; alignment control group, AC) using DESeq2. To better discern effects of *Cal-zld* knockdown on the initiation of zygotic transcription, we distinguished genes with maternal expression (227), genes with maternal and zygotic expression (6658), and genes with only zygotic expression (691) (Fig 3, S4 Appendix). As expected, only very few maternal genes were affected by knockdown of *Cal-zld*. In contrast, genes with maternal and zygotic expression were predominantly upregulated (1.7-fold in zldKD-vs-AC; 2.1-fold in zldKD-vs-zldnoKD) while genes with strictly zygotic expression were predominantly downregulated (3.3-fold in zldKD-vs-AC; 13.7-fold in zldKD-vs-zldnoKD). While the predominant upregulation of many genes with maternal and zygotic expression could reflect indirect posttranscriptional roles of *Cal-zld* in their regulation, the predominant downregulation of strictly zygotic genes in response to reduced *Cal-zld* levels is consistent with an important role of Cal-Zld in their transcriptional activation.

**Fig 4:**
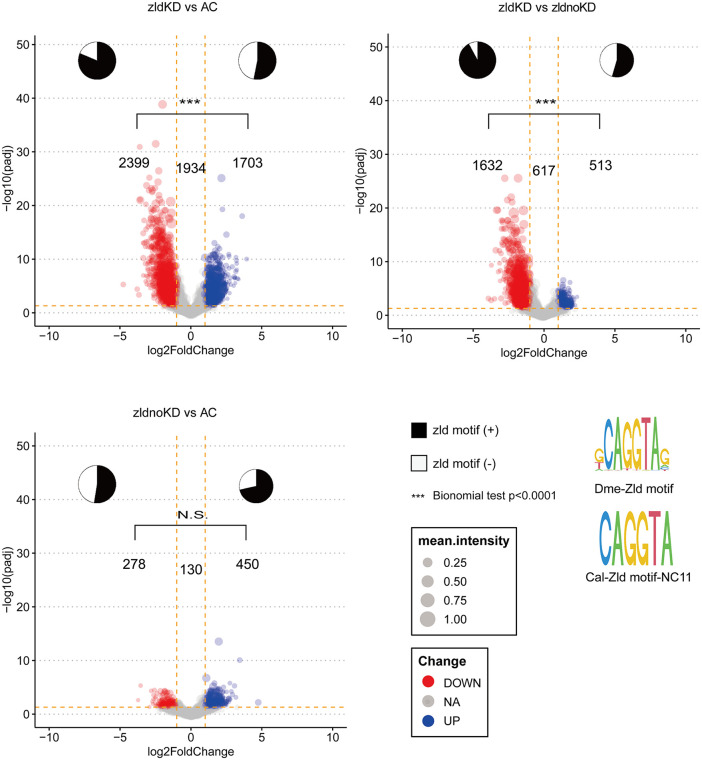
Cal-Zld promotes chromatin accessibility. Volcano plots of differentially accessible chromatin regions (ATAC-seq peaks) between *Cal-zld* RNAi embryos with efficient *Cal-zld* knockdown and alignment controls (zldKD vs. AC) or *Cal-zld* RNAi embryos without *Cal-zld* knockdown (zldKD vs. zldnoKD). The comparison of *Cal-zld* RNAi embryos without *Cal-zld* knockdown to alignment controls (zldnoKD vs. AC) serves as a negative control. Significant change of adjusted *p*-value ≤ 0.05 and |log_2_ fold change| ≥ 1 are highlighted in red (log_2_ fold change ≤ −1) or blue (log_2_ fold change ≥ 1). The inserted pie charts show the proportion of peaks with at least one Zld motif (black) as reported in FlyFactorSurvey or inferred from the discriminative MEME analysis on NC11 peaks in wild-type Clogmia embryos (cf. position weight matrix logos). Underlying data is available in S3 Data and NCBI (bioproject: PRJNA1198932).

At the level of chromatin accessibility, (Figs 4, S7, S5 Appendix), 4,102 regions (12.8% of all 32,157 ATAC-seq peaks) were affected in the zldKD-vs-AC comparison, and 2,145 peaks (8.6% of all peaks) were affected in the zldKD-vs-zldnoKD comparison. In both cases, the majority of the peaks lost accessibility (1.4-fold difference in zldKD-vs-AC; 3.2-fold difference in zldKD-vs-zldnoKD; 5.7-fold differences between consistently downregulated and upregulated peaks). These observations should be compared to our negative control (zldnoKD-vs-AC), in which only 2.3% of all peaks exhibited differential accessibility and no enrichment of downregulated peaks was observed (Fig 4). Depletion of *Cal-zld* mRNA affected both dynamic peaks and nondynamic peaks. Dynamic peaks that lost accessibility in response to *Cal-zld* knockdown mostly belonged to groups that opened before late NC14 (e.g., groups 8, 15, 14, 12, 6, 10; S8 Fig). Taken together, these findings suggest that Cal-Zld plays an important role in opening chromatin while a limited additional role in closing select chromatin regions cannot be ruled out.

To assess if the changes in accessibility were a direct or indirect consequence of *Cal-zld* knockdown, we performed two types of motif analyses. First, we performed a differential motif enrichment analysis with MEME on *Cal-zld* sensitive peaks to identify motifs enriched centrally within 50 bp of the peak summit (S9 Fig). Relative to all nonsensitive peaks, the Zld motif was enriched in peaks that lost accessibility following *Cal-zld* knockdown (zldKD versus AC: 5-CAGGTA, E = 1.9e^−76^; zldKD versus zldnoKD: 5-CAGGTA, E = 1.9e^−89^), consistent with a direct role of Cal-Zld in nucleosome depletion and chromatin opening. Conversely, the Zld motif was not enriched in peaks that gained accessibility. We then extended the search for Cal-Zld motifs to the full width of each peak, using Zld motifs derived from our MEME analysis in Clogmia and experimental data in *Drosophila melanogaster* [116]. 81.4% of the 2,399 downregulated peaks in zldKD-vs-AC and 92.1% of the 1,632 downregulated peaks in zldKD-vs-zldnoKD contained at least one Zld motif compared to ~60% of the respective upregulated peaks or the Cal-Zld-insensitive peaks. This difference was slightly more pronounced when restricting the comparison to the 1,091 consistently downregulated peaks (93.3% with at least one Zld motif; S7A Fig) and the 191 consistently upregulated peaks (47.1% with at least one Zld motif; S7B Fig). These findings suggest that Cal-Zld plays a direct role in opening chromatin. In support of this hypothesis, we recall that both motif affinity and occupancy positively correlate with Zld’s pioneering activity in Drosophila [54,109]. Conversely, the closure of many chromatin regions that we observed during the final cleavage cycles in wild-type Clogmia embryos may generally occur without direct Cal-Zld input, although a direct role in closing some of these regions cannot be ruled out since 90 of the 191 consistently upregulated peaks in the zldKD-vs-AC and zldKD-vs-zldnoKD comparisons contain at least one Zld motif.

### Maternal Cal-Opa drives chromatin accessibility and expression at *homeobrain* and *sloppy-paired* loci in complementary anterior domains

The early zygotic segmentation gene network of dipterans is dominated by TF-encoding genes, such as the gap genes and pair-rule genes [20,22,30,80,117–120]. Bcd breaks axial symmetry by targeting these genes in a concentration-dependent manner throughout the blastoderm stage, including NC14. In contrast, maternal *Cal-opa* exerts its symmetry-breaking function in axis specification prior to the onset of zygotic *Cal-opa* expression during blastoderm cellularization at mid NC14 [3]. Therefore, we focused our search of Cal-Opa targets on TF genes that are expressed before NC14 and asked whether maternal *Cal-opa* activity affects their expression and chromatin accessibility during NC12 and NC13.

**Fig 5:**
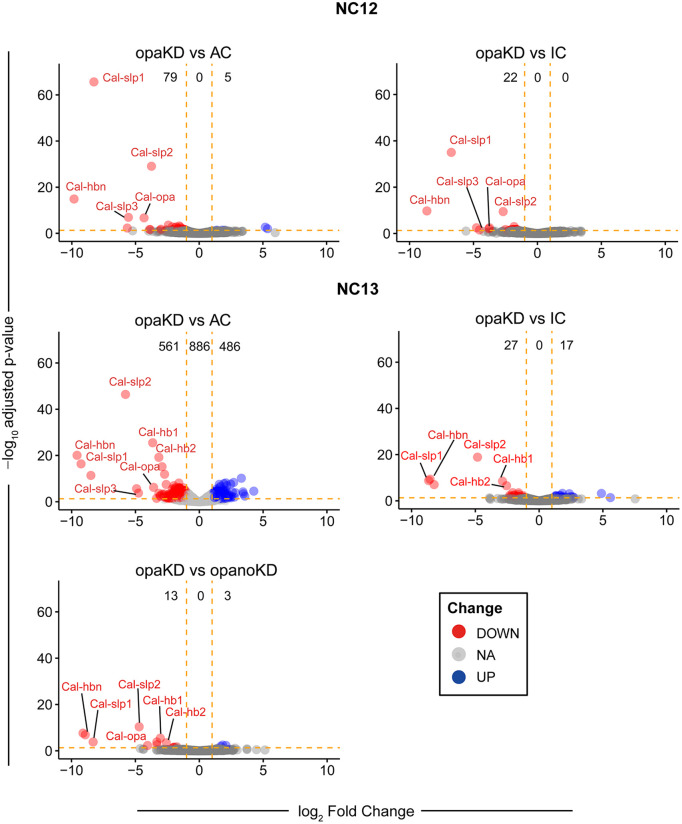
Maternal *Cal-opa* activates a small set of transcription factor genes at NC12 and NC13. Volcano plots of differentially expressed genes between embryo groups with reduced *Cal-opa* expression (opaKD) and control groups (AC and IC for NC12; AC, IC, and opanoKD for NC13). See S11A Fig for a negative control (opanoKD vs. AC at NC13). Significant changes of adjusted *p*-value ≤ 0.05 and |log_2_ fold change| ≥ 1 are highlighted in red (log_2_ fold change ≤ −1) and blue (log_2_ fold change ≥ 1). Consistently affected TF genes are labeled. Underlying data is available in S4 Data and NCBI (bioproject: PRJNA1198932).

**Fig 6:**
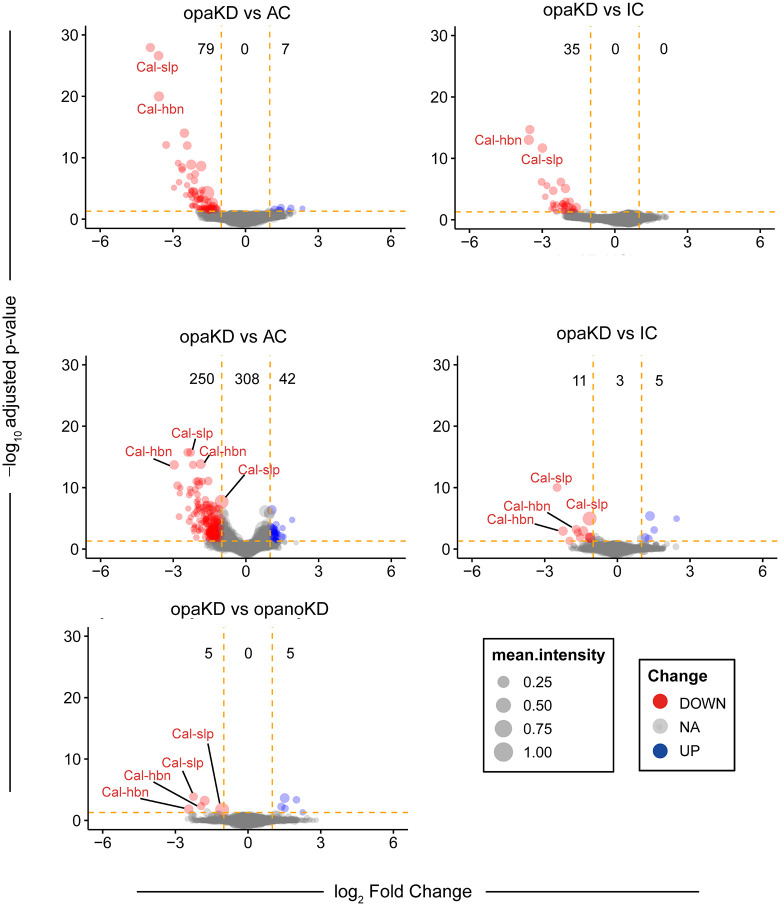
Cal-Opa is required for establishing a selective set of chromatin accessibility peaks. Volcano plots of differentially accessible chromatin regions (ATAC-seq peaks) between *Cal-opa* RNAi embryos with reduced *Cal-opa* expression (opaKD) and control groups (AC and IC for NC12; AC, IC, and opanoKD for NC13). See S11B Fig for negative control (opanoKD vs. AC). Significant changes of adjusted *p*-value ≤ 0.05 and |log_2_ fold change| ≥ 1 are highlighted in red (log_2_ fold change ≤ −1) and blue (log_2_ fold change ≥ 1). Underlying data is available in S5 Data and NCBI (bioproject: PRJNA1198932).

To assess the impact of maternal Cal-Opa activity on gene expression and chromatin accessibility, we performed RNA-seq and ATAC-seq on single *Cal-opa* RNAi embryos (6 replicates for NC12, 8 replicates for NC13) and selected four NC12 embryos and five NC13 embryos with knockdown efficiencies greater than 75% (NC12_opaKD, NC13_opaKD; S10 Fig and Materials and methods). At NC13, we also identified two RNAi embryos without *Cal-opa* knockdown (NC13_opanoKD) and used them as a negative control group. In parallel, we processed stage-matched embryos that were prepared for injection under oil but not injected (alignment controls, AC; 4 replicates for each developmental stage), and embryos that were injected with dsRNA of the extraneous gene *DsRed* (injection controls, IC; 3 replicates for each stage). We then used DESeq2 to compare gene expression (RNA-seq peaks; Fig 5, S6 Appendix) and chromatin accessibility (ATAC-seq peaks; Fig 6, S7 Appendix) at NC12 and NC13 between embryos of the opaKD groups and their respective control groups (AC and IC for NC12; AC, IC, and opanoKD for NC13). At NC12, opaKD-vs-AC identified 79 downregulated genes (including 11 TF genes) and 5 upregulated genes (no TF genes) while opaKD-vs-IC identified only 22 downregulated genes (including 5 TF genes) and no upregulated genes. An opanoKD group was not available for this stage. *Cal-opa* (positive control), Clogmia’s three *sloppy-paired* homologs (*Cal-slp1, Cal-slp 2, Cal-slp3*), and *homeobrain* (*Cal-hbn*) were the only consistently downregulated TF genes at NC12 (Fig 5). At NC13, the opaKD-vs-AC comparison yielded 561 downregulated genes (including 67 TF genes) and 486 upregulated genes (including 49 TF genes). However, far fewer down- and upregulated genes were identified when the opaKD group was compared to the injected control groups (Fig 5). In opaKD-vs-IC, 27 genes were downregulated (including 5 TF genes: *Cal-hbn, Cal-slp 1, Cal-slp2, Cal-hb1, Cal-hb2*) and 17 were upregulated (including 1 TF gene). Downregulation of *Cal-opa* in the IC control group was not statistically significant (log_10_ adjusted *p*-value of 0.1169), falling slightly above our significance cutoff: log_10_ adjusted *p*-value of < 0,05. This observation likely reflects the low detection of *Cal-opa* transcript in one of the three embryos in the injection control group (S10 Fig). In opaKD-vs-opanoKD, only 13 genes were downregulated (including 6 TF genes: *Cal-hbn, Cal-slp1, Cal-slp2, Cal-hb1, Cal-hb2,* and *Cal-opa*) and 3 genes were upregulated (0 TF genes). These results should be compared to our negative control for NC13 (opanoKD-vs-AC), which only yielded a random set of 3 slightly downregulated genes (0 TF genes) and no upregulated genes (S11A Fig). Taken together, our results suggest that maternal *Cal-opa* promotes the activation of *Cal-slp1, Cal-slp 2, Cal-slp3*, and *Cal-hbn* at NC12 and of *Cal-slp1*, *Cal-slp2, Cal-hbn*, *Cal-hb1,* and *Cal-hb2 at* NC13. Of these genes, only the loci of *Cal-slp1, Cal-slp 2,* and *Cal-hbn* were associated with ATAC-seq peaks that spanned Opa motifs (S7 Appendix).

**Fig 7:**
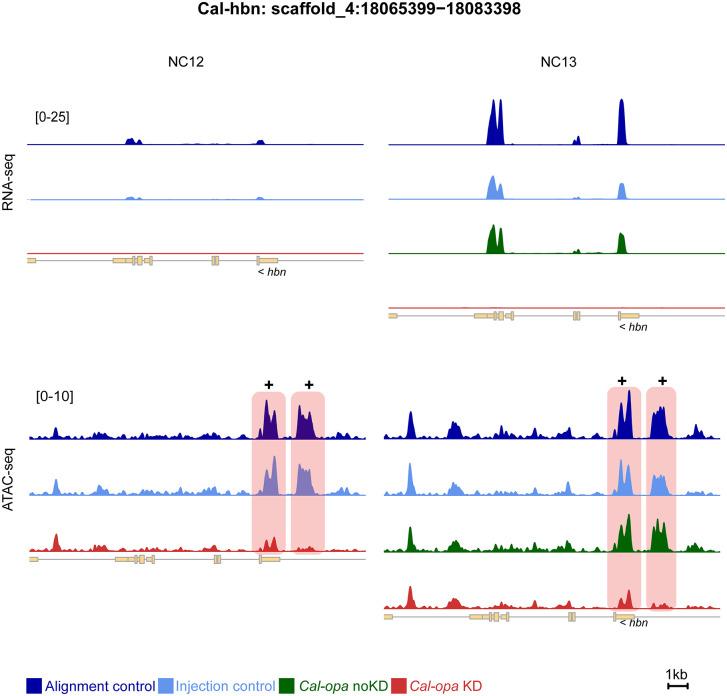
Expression and chromatin accessibility at the *Cal-hbn* locus at NC12 and NC13. RNA-seq and ATAC-seq tracks (CPM) are shown for NC12 and NC13 with alignment control in blue, injection control in light blue, *Cal-opa* RNAi without KD in green, and *Cal-opa* RNAi with KD in red. Gene models are shown in pale yellow. ATAC-seq peaks with significant changes in accessibility are highlighted in pink and are marked with a plus sign if they contain at least one Opa motif. The scale of the y-axis is adjusted for each data type but kept constant between stages and is indicated with numbers in square brackets: [0-25] for RNA-seq data; [0-10] for ATAC-seq data. Underlying data is available in NCBI (bioproject: PRJNA1198932).

**Fig 8:**
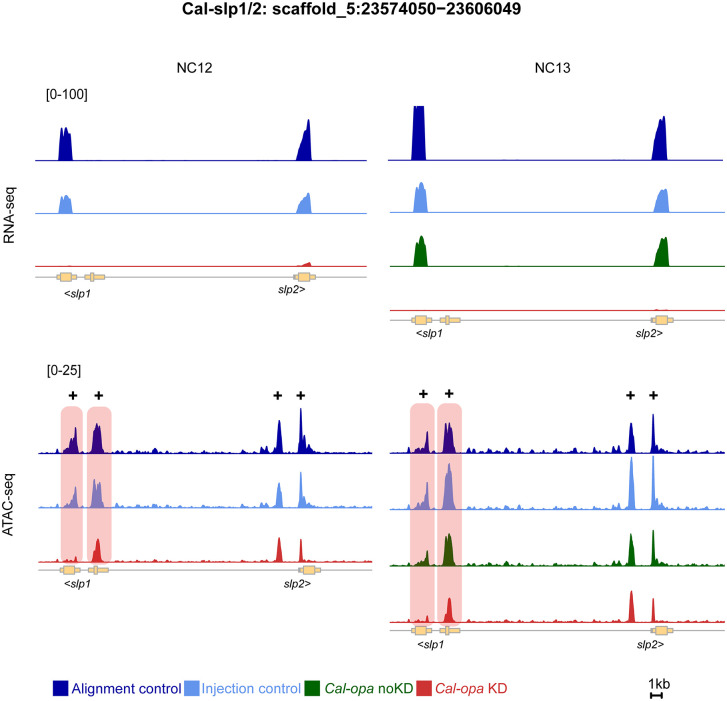
Expression and chromatin accessibility at the *Cal-slp1 and Cal-slp2* locus at NC12 and NC13. RNA-seq and ATAC-seq tracks are shown for NC12 and NC13 with alignment control in blue, injection control in light blue, *Cal-opa* RNAi without KD in green, and *Cal-opa* RNAi with KD in red. Gene models are shown in pale yellow. ATAC-seq peaks with significant changes in accessibility are highlighted in pink and are marked with a plus sign if they contain at least one Opa motif. The scale of the y-axis (CPM) is adjusted for each data type but kept constant between stages and is indicated with numbers in square brackets: [0-100] for RNA-seq data; [0-25] for ATAC-seq data. Underlying data is available in NCBI (bioproject: PRJNA1198932).

To assess the effects of *Cal-opa* knockdown on chromatin accessibility we performed DESeq2 analyses on the ATAC-seq peaks (Fig 6, S7 Appendix). At NC12, the opaKD-vs-AC comparison identified 79 downregulated and 7 upregulated peaks. Sixty-three of the 79 peaks downregulated peaks but none of the 7 upregulated peaks contained at least one Opa motif. The opaKD-vs-IC comparison only yielded 35 downregulated peaks of which 30 contained at least one Opa motif. The 32 consistently downregulated peaks were associated with the loci of *Cal-slp1*, *Cal-hbn, Cal-opa,* and various non-TF genes (Figs 7, 8, S7 Appendix). One additional ATAC-seq peak associated with *Cal-slp2* narrowly missed our significance threshold in the opaKD-vs-IC comparison (adjusted *p*-value 0.062).

At NC13, opaKD-vs-AC yielded 250 downregulated peaks and 42 upregulated peaks, OpaKD-vs-IC identified 11 downregulated peaks and 5 upregulated peaks, and opaKD-vs-opanoKD recovered only 5 downregulated peaks and 5 upregulated peaks without Opa motifs (Fig 6, S7 Appendix). Of the consistently downregulated peaks, two were associated with *Cal-hbn* (Fig 7), and two were associated with *Cal-slp1* (Fig 8), all covering Opa motifs. The fifth consistently downregulated peak was associated with an uncharacterized gene without homology to known genes (evm.TU.scaffold_7.1426) and did not cover an Opa motif. Significant downregulation of peaks associated with *Cal-hb1* and *Cal-hb2* was only observed in comparisons with AC and IC controls at NC13, and no Opa motifs were detected in these peaks (S12 Fig). In our negative control (opanoKD-vs-AC), none of these ATAC-seq peaks were recovered (S11B Fig, S7 Appendix).

**Fig 9:**
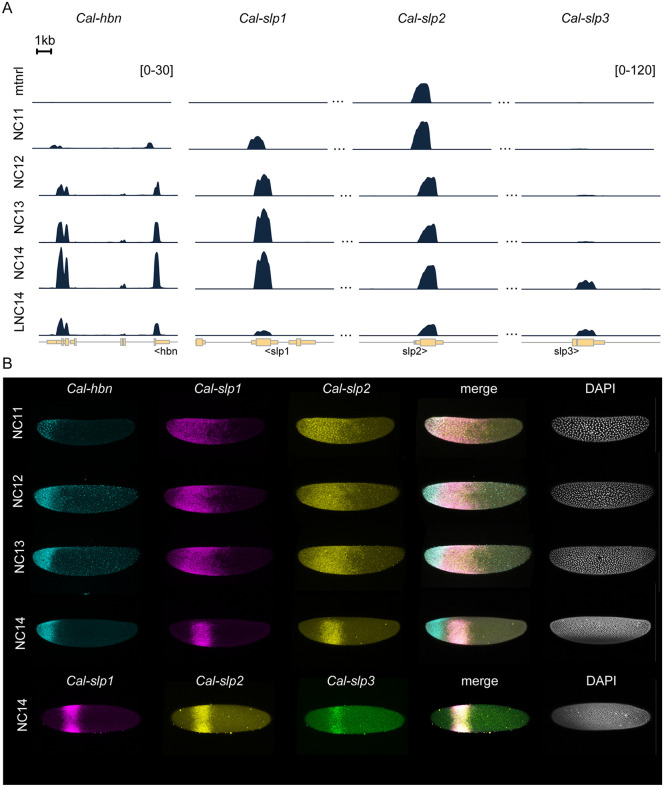
Expression of *homeobrain* and *sloppy-paired* homologs in Clogmia. **A)** Expression levels (CPM) of *Cal-hbn*, *Cal-slp1*, *Cal-slp2*, and *Cal-slp3* at 6 consecutive stages, including NC2/3 (maternal, ~45-min old embryos), NC11, NC12, NC13, NC14 and Late NC14. Gene models are shown in pale yellow. Synteny of the three sloppy-paired loci is indicated by three dots. The y-axis is the same for all tracks at each locus. *Cal*-*hbn* RNA-seq: 0-30 CPM. *Cal*-*slp1-3* RNA-seq: 0-120 CPM. **B)** Whole-mount fluorescent in situ hybridizations at consecutive blastoderm stages. Embryos are shown with anterior left and dorsal up. Underlying data is available in NCBI (bioproject: PRJNA1198932).

Given that maternal *Cal-opa* is required for zygotic transcription *homeobrain* and *sloppy-paired* homologs, we examined the expression of these genes in more detail. Maximum-likelihood trees with dipteran and nondipteran *sloppy-paired* homologs generated with IQ-TREE v.2.3.6. [121] and ModelFinder [122] suggest that *Cal-slp1, Cal-slp2,* and *Cal-slp3* resulted from recent gene duplications in the Clogmia lineage (S13, S14 Figs). Zygotic transcription of Clogmia’s *sloppy-paired* and *homeobrain* homologs was detected during and after NC11 and was confined to the anterior half of the blastoderm (Fig 9). *Cal-hbn* was expressed in the anterior-most portion of the blastoderm, complementary to the overlapping expression domains of *Cal-slp1*, *Cal-slp2*, and *Cal-slp3* (which was only expressed at low levels prior to NC14)*.* In the case of *Cal-slp2,* we also detected high levels of maternal transcript, previously found to be distributed in a shallow anterior-to-posterior gradient [3]. The early timing and spatial expression of Clogmia’s *homeobrain* and *sloppy-paired* homologs suggest that these genes likely play an important zygotic role in the initiation of anterior pattern formation.

## Discussion

### Dynamic chromatin states in the embryo of *Clogmia albipunctata*

In this study, we have established the resources for dissecting developmental gene networks of early Clogmia embryos by providing an annotated chromosome-level genome sequence for our inbred laboratory strain and by describing chromatin accessibility of precisely staged consecutive blastoderm stages. We have used these resources to examine how the activities of *zelda* and maternal *odd-paired* shape chromatin accessibility and gene expression in Clogmia. We found that, as in Drosophila, the period spanning ZGA is accompanied by large-scale changes in chromatin accessibility. However, there are notable differences between Clogmia and Drosophila in the early patterns of establishment and maintenance of accessible states before NC14. In Clogmia, the chromatin landscape is highly dynamic in that gains as well as losses occur during the nuclear cleavage cycles of the blastoderm stage. In Drosophila, only gains in accessibility are observed until widespread ZGA and lengthening of the cell cycle at NC14 [74,76,77,79,123]. This difference could be a consequence of cell cycle timing. In Clogmia, NC11 through NC13 last ~3x longer than in Drosophila [83], in which NC11 only lasts ~10 min, NC12 ~12 min, and NC13 ~21 min [82]. The much shorter cell cycle times of Drosophila present significant limitations to establish and maintain chromatin states necessary for transcriptional activity [79]. Assuming that kinetic rates for nuclear import, DNA replication, and DNA binding of TFs is similar between Clogmia and Drosophila, the significantly longer syncytial interphase times in Clogmia could alleviate constraints on the initiation of zygotic transcription. However, the timing of AP patterning between the two species appears comparable [84,117], suggesting that the timing of ZGA might also be conserved. Alternatively, the longer syncytial interphase times in Clogmia could alleviate constraints on maintaining or silencing active chromatin states. In Drosophila, Polycomb group (PcG) proteins begin to establish heritable, transcriptionally silenced chromatin states during NC14 [124], but in Clogmia, the longer nuclear cleavage cycles may provide time for the maturation of repressive histone marks at accessible chromatin regions before NC14.

### Role of Zld in regulating chromatin accessibility during Clogmia’s nuclear cleavage cycles

We found that reduced *Cal-zld* expression results in widespread losses of chromatin accessibility, consistent with a conserved role for Zld to pioneer chromatin accessibility within the context of ZGA. In Drosophila, Zld almost exclusively drives gains in chromatin accessibility during nuclear cleavage cycles [71,76,102,125,126]. However, two recent studies found that during ZGA, Zld activity directs how repressive chromatin marks are deposited. Loss of *zld* results in context-specific losses and gains of H3K27me3 within specific PcG domains [124]. Zld also enables histone acetylation at promoters and enhancers through its interaction with Chip, Drosophila’s homolog of LIM-domain-binding protein 1 (Ldb1), which in turn recruits the histone acetyltransferase CBP [127]. The longer nuclear cleavage cycles in Clogmia might enable similar roles of Cal-Zld before NC14. In this case, Clogmia and Drosophila may differ in the timing of when specific histone marks are deposited and affected by Zld depletion. Differences in the activities of Zld and Cal-Zld could also reflect differences in their protein structures or isoforms. For example, Cal-Zld contains an additional zinc finger that has been eroded in Drosophila (S4 Fig) [110]. Additionally, stage-specific isoforms of Zld have been described in Drosophila, including a variant lacking the C-terminal three zinc fingers [128–131]. This isoform functions as a dominant negative form in cell culture although flies in which the expression of this isoform is suppressed are viable and fertile [130]. Therefore, the molecular underpinnings of the functional readouts of Zld and Cal-Zld could differ and involve both direct and indirect mechanisms.

### Functional comparison of the anterior determinants in Clogmia and Drosophila

Bcd contributes to the activation of many early segmentation genes; however, its role in the posterior embryo is much more subtle than in the anterior embryo, where all expression domains directly or indirectly depend on Bcd. Bcd target enhancers have been classified based on how their accessibility depends on Bcd and Zld [71]. The accessibility of Bcd target enhancers that drive expression in the posterior of the embryo is established independent of Bcd (e.g., *Krüppel* CD1, *knirps* posterior, *giant* posterior). However, enhancers that open in a Bcd-dependent manner are associated with genes expressed in the anterior of the embryo (e.g., *orthodenticle*, *empty spiracles*, *buttonhead*, *giant*, *knirps*, *hunchback*, *sloppy-paired 1*, *fd19B*, *even-skipped*, *paired*, *cap’n’collar*, *huckebein, and homeobrain*). Moreover, many correspond to enhancers that drive reporter gene expression in the anterior embryo including *giant* anterior (−6), *giant* anterior (−10), *knirps* anterior, *orthodenticle*, *buttonhead, even-skipped* stripe 1/5, *hunchback* P2, *hunchback* shadow enhancers, *eve-skipped* stripe 2*,* an early *paired* enhancer, *cap’n’collar*, and *huckebein* (reviewed in [71]). The majority of Bcd-dependent anterior expression domains first appear during NC13, with the noteworthy exception the P2 transcript of *hunchback*. The promoter of this transcript is in an open conformation as early NC10 [79] and drives Bcd-dependent expression throughout the anterior half starting at NC11 [45,66,132], thereby boosting the maternal Hb gradient which forms independent of Bcd [133].

In Clogmia, neither the expression nor chromatin accessibility at the *Cal-hb1* and *Cal-hb2* loci was reduced in NC12 opaKD embryos (S12 Fig) and maternal *hunchback* activity does not contribute to embryo polarity in this species, given that knockdown of maternal *Cal-opa* transcripts results in symmetrical double abdomens [3]. Additionally, homologs of several Bcd target genes were not expressed at NC12 and NC13 (e.g., *Cal-gt*, *Cal-kni/knrl1-2, Cal-ems1-4*, and *Cal-hkb*) or their expression levels at these stages were not affected by the knockdown of maternal *Cal-opa* activity (e.g., *Cal-cnc, Cal-prd, and Cal-btd*). However, maternal Cal-Opa was required for the zygotic transcription of *sloppy-pai*red and *homeobrain* homologs in complementary, evolutionarily conserved domains across the anterior half of the embryo. In Drosophila, these genes are regulated by Bcd-bound enhancers whose accessibility is sensitive to the concentration of Bcd [134,135], even though neither of them has received much attention as Bcd target genes. Taken together, these findings suggest that the anterior determinant of Clogmia targets a small subset of the genes known as direct targets of Bcd in Drosophila.

Mechanistically, Cal-Opa could prime accessibility at target loci through a canonical pioneering mechanism which requires binding of nucleosome occupied DNA and promoting nucleosome instability [47,48,56,60,136,137]. A vertebrate homolog of Opa, Zic3, was found to bind nucleosomal DNA in vivo, suggesting that Cal-Opa may pioneer chromatin directly [138]. Alternatively, Cal-Opa binding may compete with nucleosomes to bind unoccupied DNA during each nuclear cleavage cycle [139], similar to Bcd [54,71,78].

### Role of *homeobrain* and *sloppy-paired* in breaking axial symmetry in insects

It is conceivable that *homeobrain* nor *sloppy-paired* homologs serve a more broadly conserved role in the initiation of anterior pattern formation. In Drosophila, *homeobrain* mutant embryos lack the labrum and anterior portions of the brain [140–143], indicating that *homeobrain* is essential for specifying the most anterior portion of the embryo. A more extreme *homeobrain* loss-of-function phenotype has been observed in the beetle *Tribolium castaneum*, where this gene plays an important zygotic role in establishing the embryo’s AP polarity [144]. In Tribolium, knockdown of *homeobrain* resulted in loss of the entire head and—in extreme cases—duplications of the tail end. The duplicated abdomen was defective, and thoracic structures persisted in these embryos, but complete double abdomens could be obtained by simultaneously suppressing the expression *Tc-zen1* (like *bicoid* a *Hox3* derivative [35,95], thereby preventing the anterior repression of *caudal*, an important gene for posterior patterning [145]. Additionally, the Tribolium ortholog of *odd-paired*, which is expressed maternally and required for head development (including the labrum, antennae, and head capsule) [146], could potentially complement the strictly zygotic roles of *Tc-zen* and *Tc-hbn* in breaking symmetry along the AP axis of the Tribolium embryo.

The *sloppy-paired* gene, a member of the *forkhead* domain gene family [147], is best-known for its role as a pair-rule segmentation gene [22,81], but it is initially expressed in a head domain of the syncytial Drosophila embryo, along with its paralogs *sloppy-paired 2* and *fd19B* [135,148–153]. The *sloppy-paired* paralogs, which are common across insects (S13, S14 Figs), may have obscured a fundamental role of *sloppy-paired* activity in initiating anterior pattern formation in Drosophila. This also applies to the two *sloppy-paired* homologs of Tribolium, in which only the gnathocephalon (mandibular, maxillary, and labial segment) and even-numbered trunk segments are lost when *Tca-slp1* is knocked down [154,155]. We therefore propose that along with *homeobrain*, *sloppy-paired* may play a more widely conserved role in establishing the AP axis in insect embryos.

### Limitations of the study

It is conceivable that *homeobrain* and *sloppy-paired* activities are sufficient for breaking symmetry along the AP axis of the Clogmia embryo. To test this hypothesis, it might be necessary to completely suppress the activities of four genes, including three with early zygotic expression (*Cal-hbn*, *Cal-slp1*, and to a lesser degree *Cal-slp3*) and one with anterior-enriched maternal transcript and zygotic expression (*Cal-slp2*). This is particularly challenging in Clogmia because this species did not tolerate more than one microinjection per embryo in our hands. Ongoing research in our laboratory seeks to overcome this technical issue by developing more effective knockout and knockdown reagents. Alternatively, maternal Cal-Opa could control the zygotic expression of additional genes. We did not observe other TF genes that were consistently downregulated in embryos with reduced maternal *Cal-opa* transcript, but this could be due to incomplete knockdown of the maternal *Cal-opa* transcript or limited statistical power of our opaKD-vs-IC and/or opaKD-vs-opanoKD comparisons or a combination of both. To further explore this possibility and to formally prove a direct interaction of maternal Cal-Opa with the identified target genes, we are developing protocols and reagents for Cal-Opa chromatin immunoprecipitation experiments. In the present study, the identification of Clogmia’s anterior determinant target genes rests on Opa-dependent chromatin accessibility along with the presence of Opa-binding motifs, Opa-dependent gene expression in the anterior blastoderm, and the early timing of transcriptional activation.

## Materials and methods

### Clogmia culture and embryo collection

The Clogmia culture was maintained in 100 mm × 15 mm Petri Dishes (Fisher Scientific FB0875712) on moist, compressed cotton (Jorvet SKU: J0197) sprinkled with parsley leaf powder (Starwest Botanicals SKU: 209895-5). For embryo collections, ~2-day old females were transferred to round 250 mL glass bottles with a ~1-inch bottom layer of moistened and compressed cotton and mated at room temperature for 2–3 days. Female ovaries were dissected with a pair of fine forceps (Dumont 5) and eggs released into water for egg activation and embryogenesis. For staged egg collections, embryos were released into flat bottom glass 100 mm × 15 mm Petri Dishes (Corning CM: 101425,55) containing 15 ml of water. Care was taken to disperse the eggs on the bottom of the dish as crowding can delay development. Petri Dishes were covered with a lid and moved to an incubator set at 25 °C until the desired stage. Egg harvesting post activation was on average 47 min for NC2/3, 261 min for NC11, 292 min for NC12, 332 min for NC13, 401 min for NC14, and 465 min for Late NC14.

### Embryo fixation

Embryos were dechorionated using a 50% dilution of commercial bleach (8.25% sodium hypochloride) for 60 s. Dechorionated embryos were fixed in a 50 mL falcon tube, using 20 mL of boiling salt/detergent-solution (100 μL 10% triton-X; 500 μL 28% NaCl; up to 20 mL of water). The mixture was gently swirled for 10 s. To stop the heat fixation, 30 mL of deionized water was added. Embryos were transferred to 1.5 mL reaction vials and devitellinized in a 1:1 mixture of n-heptane and methanol by vigorous shaking. This step was followed by 3 washes with cold methanol (stored at −80 °C). Embryos were inspected under a stereomicroscope and manual devitellinizations were performed as needed using sharp tungsten needles in a 3% agar plate covered with methanol. Devitellinized embryos were stored in 100% methanol at −20 °C.

### Microinjections of embryos and RNAi efficiency

Embryo injection was done as previously described [3]. We note here that RNAi efficiency in Clogmia embryos varies [3]. This variability could be due to technical variables (e.g., injection needle, amount injected) or biological variables (e.g., differences in the RNAi response between individual embryos, or the general health or genetic background of the embryos or their mothers) or a combination of such effects. Therefore, we took care to control the age of the females and selected all experimental embryos from females with healthy looking ovaries and eggs. Embryos were injected within the first hour of development (NC2/3). To test *Cal-zld* dsRNA, we quantified knockdown of the *Cal-zld* transcript in individual embryos by RT-qPCR and performed fold change calculations of *Cal-zld* mRNA levels with the average level in uninjected control embryos as reference (S3 Table). In one quarter of these *Cal-zld* RNAi embryos (5/20), *Cal-zld* mRNA levels were reduced by ~30%–90%. For *Cal-opa* RNAi, we used dsRNA of the isoform-specific first exon of the maternal *Cal-opa* transcript and confirmed that it can induce the double abdomen phenotype. We also quantified knockdown efficiencies of the *Cal-opa* transcript in individual embryos by RT-qPCR and performed fold change calculations of *Cal-opa* mRNA levels with the average level in uninjected control embryos as reference (S4 Table). This analysis suggests that *Cal-opa* RNAi can nearly abolish the *Cal-opa* transcript.

### dsRNA synthesis

A PCR template was made using primers specific for the target gene with T7 overhangs and either cDNA, gDNA, or a plasmid depending on the target sequence. The PCR product was run on a gel and purified using the Zymo Gel DNA Recovery Kit (D4007/D4008) following the manufactures instructions. The PCR template was used as the input for the transcription reaction producing dsRNA. dsRNA synthesis and purification were done using the Invitrogen MEGAscript RNAi Kit (AM1626) following the manufacturer’s instructions. Purified dsRNA was eluted in injection buffer (0.1 mM NaH_2_PO_4_ Ph 6.8; 5 mM KCl).

Forward and reverse primer sequences for dsRNA (lengths of dsRNAs in brackets; gene specific sequence of primers underlined):

*Cal-opa,* maternal transcript (201 bp):

5′-CAGAGATGCATAATACGACTCACTATAGGGAGAAAACAATTGTGAAGTGCGACA

5′-CAGAGATGCATAATACGACTCACTATAGGGAGACAAATTTCCAAACGATGACAGA

*Cal-zld* (a combination of two dsRNAs were used 579 bp and 668 bp):

5′-TAATACGACTCACTATAGGGAGAAGTCCCGCAATTGATACAGC

5′-TAATACGACTCACTATAGGGAGAAGGATGTTGGTGGACACTCC

5′-TAATACGACTCACTATAGGGAGACGGAATGGTGTGTGAAACAG

5′-TAATACGACTCACTATAGGGAGATTCGAGGGGTTATTGTCCTG

dsRed (273 bp):

5′-CAGAGATGCATAATACGACTCACTATAGATGCAGAAGAAGACTATGG

5′- CAGAGATGCATAATACGACTCACTATAGCTACAGGAACAGGTGGTG

### RT-qPCR

RT-qPCR reactions were performed using the Luna Universal One-Step RT-qPCR Kit (New England Biolabs Ipswich, MA).

Forward and reverse primer sequences for targets (lengths of amplicon in brackets):

*Cal-opa*, maternal transcript (112 bp):

5′- TTGCGCTTCATTCTGTCATCG

5′- CACTGAGGGCTGATTCTCGG

*Cal-zld* (138 bp):

5′-AAAGATGAACCACCGTGCGA

5′-CATTGATGGCAGTGGACCCT

*Cal-rpl35* (119 bp):

5′- CTGTCCAAAATCCGAGTCGT

5′- AGGTCGAGGGGCTTGTATTT

### Fluorescent HCR in situ hybridizations

Gene specific probes and amplifier-fluorophore sets were designed and synthesized by Molecular Instruments Inc (Los Angeles, CA). The amplifier-fluorophores were used as follows: B1-647 for *Cal-slp1*, B2-546 for *Cal-hbn* and *Cal-slp3*, and B3-488 for *Cal-slp2,* and *Cal-otd1*. Buffers (probe hybridization, probe wash, and amplification buffers) throughout the protocol were purchased from Molecular Instruments. The HCR in situ hybridizations were performed as described [156,157]. Briefly, fixed embryos were rehydrated and washed 3 times in PBS-Tween20 (1x PBS, 0.1% Tween-20). Embryos were permeabilized for 30 min at room temperature (100 ml Permeabilization buffer: 100 µL 100% Triton X-100, 50 µL Igepal CA-630, 50 mg Sodium Deoxycholate (Sigma D6750-25G), 50 mg Saponin (Sigma 47036-50G-F), and 200 mg BSA Fraction V (Sigma A5611-5G). Embryos were then pre-hybridized in probe hybridization buffer for 30 min in a 37 °C water bath. Once pre-hybridization was complete, fresh hybridization solution containing gene specific probes was added, and the embryos were left to incubate overnight in a 37 °C water bath. On the second day the hybridization solution was removed, and the embryos were washed 4 times with pre-warmed probe wash buffer followed by two washes in 5x SSCT (5x SSC, 0.1% Tween-20). An amplifier hairpin solution was prepared following the manufacturer’s instructions. Embryos were incubated with pre-warmed amplification buffer for 30 min at room temperature before adding amplifier hairpin solution. Embryos were left to incubate overnight at room temperature in the dark. The following day the embryos were washed 5 times in 5x SSCT at room temperature. The embryos were then counter stained with DAPI and mounted in Aqua-Polymount for imaging.

### Image acquisition and processing

Confocal imaging was performed on the Zeiss LSM 900 using a Plan-Apochromat 20x/0.8 M27 objective. Pixel-density was 1,024 x 1,024 and physical pixel size was X = 0.46980808984799 µm and Y = 0.46980808984799 µm. Excitation wavelengths were 561 nm for Alexa Fluor 546 with B2 amplifier (*Cal-hbn/Cal-slp3*; collection window: 550–645 nm), 640 nm for Alexa Fluor 647 with B1 amplifier (*Cal-slp1*; collection window: 645–700 nm), 488 nm for Alexa Fluor 488 with B3 amplifier (*Cal-slp2*; collection window: 410–550), and 405 nm for DAPI (collection window: 410–550 nm). Z-slice spacing was 1 µm. For each image a Z-stack of images was captured from the embryo’s surface to approximately halfway through the embryo. Then a maximal projection was created from this Z-stack using FIJI/Image J. To minimize background fluorescence, the display range minimum of each channel was slightly increased in all images (0–255 scale): 18 for 541 nm, 25 for 640 nm, and 40 for 488 nm. No further adjustments to the images were made.

### ATAC-seq library preparation

Single-embryo ATAC-seq library preparations were done essentially as described previously in *Drosophila melanogaster* [76,79]. Embryos developed in an incubator at 25 °C until the desired NC. NC was confirmed by time after egg activation and the morphology was checked under water using a transmission light microscope (Zeiss Stemi 2000-C). Once at the desired NC individual embryos were placed in the lid of a 1.5 ml low-retention Eppendorf tube and homogenized in 10 μl of cold lysis buffer (10 mM Tris-HCl pH 7.4; 10 mM NaCl; 3 mM MgCl2; 1% Igepal CA-630) [158], using a fire-sealed microcapillary tube (Drummond Microcap 25). Once homogenized, 40 µl of lysis buffer was added to the lid, and the body of the tube was carefully closed over the lid. Samples were placed on ice until all embryos were processed. The nuclei were pelleted by centrifugation at 800 RCF at 4 °C for 10 min. Supernatant removal was observed under a stereo microscope to ensure that the nuclei pellet was not dislodged. The nuclear pellet was resuspended in 7.5 µl of Tagmentation buffer (Illumina Tagment DNA TDE1 Enzyme and Buffer Kit) and placed on ice until all samples were resuspended. Then 2.5 µl of Tn5 Transposase was added and mixed by pipetting up and down. Tagmentation and amplification of ATAC-seq libraries were performed as described previously [159]. The tagmentation reaction was performed at 37 °C and 800 RPM for 30 min using an Eppendorf Thermomixer. Immediately after this step, the reactions were purified using the Qiagen Minelute kit following the manufacturer’s instructions. Purified tagmented DNA was eluted in 10 µl of buffer EB. Library amplification was performed as described previously [159]. Typically, 12–14 total PCR were performed. Amplified libraries were purified using 1.8x Ampure SpRI beads following the manufacturer’s instructions. Purified ATAC-seq libraries were eluted in 15 µl of Qiagen elution buffer (10 mM Tris-Cl, pH 8.5). The ATAC-seq library profile was evaluated using Agilent high sensitivity bio-analyzer. Concentrations were estimated using a Qubit fluorometer. Libraries from the same experiment were pooled and sequenced together. Sequencing was done at the University of Chicago Genomics Core (Chicago, IL, USA) using the Illumina NextSeq (PE 75 bp) and at Admera Health (South Plainfield, NJ, USA) using the Illumina HiSeq (PE 150 bp) and the Illumina NovaSeq X Plus (PE 150 bp).

ATAC-seq libraries were amplified using a set of modified Buenrostro primers that introduced Unique Dual Indexes (UDI) [76]. The generalized UDI ATAC primer sequences are:

ATAC UDI Index Read 2 (i5): 5′- AATGATACGGCGACCACCGAGATCTACACnnnnnnnnTCGTCGGCAGCGTCAGATGT*G -3′

ATAC UDI Index Read 1 (i7): 5′- CAAGCAGAAGACGGCATACGAGATnnnnnnnnGTCTCGTGGGCTCGGAGATG*T -3′

Primers were synthesized with a terminal phosphorothioate bond (*) by IDT (Integrated DNA Technologies, Coralville, Iowa).

### RNA isolation for RT-qPCR and RNA-seq experiments

Embryos developed in an incubator at 25 °C until the desired NC. NC was confirmed by time after egg activation and the morphology was checked under water using a transmission light microscope (Zeiss Stemi 2000-C). RNA isolation was performed using the Zymo Quick-RNA Tissue/Insect Microprep Kit following the manufacturer’s instructions except for the tissue homogenization step. Instead, homogenization was done as follows. Once at the desired NC individual embryos were placed in the lid of a 1.5 ml low-retention Eppendorf tube and homogenized in 10 μl of cold lysis buffer (10 mM Tris-HCl pH 7.4; 10 mM NaCl; 3 mM MgCl2; and 1% Igepal CA-630) [158], using a fire-sealed microcapillary tube (Drummond Microcap 25). Once homogenized the lysate was added to 450 µl of Zymo RNA Lysis Buffer and thoroughly mixed. RNA isolation resumed following the manufacturer’s instructions, including DNAse treatment. Purified RNA was eluted in DNase/RNase-Free Water.

### RNA-seq library preparation

RNA quality and quantity were assessed using the Agilent bio-analyzer. NonStrand-specific RNA-seq libraries were prepared using the Smarter v4 Ultra-Low Input protocol from Takara for cDNA synthesis and Nextera XT from Illumina for Library prep (protocols provided by Takara and Illumina). Library quality and quantity were assessed using the Agilent bio-analyzer. Libraries from the same experiment were pooled and sequenced together. Sequencing was done at the University of Chicago Genomics Core (Chicago, IL, USA) using the Illumina NovaSeq X (PE 150 bp) and at Admera Health (South Plainfield, NJ, USA) using the Illumina NovaSeq X Plus (PE 150 bp).

### Experimental setup of ATAC/RNA-seq experiments in *Cal-zld* and *Cal-opa* RNAi embryos

RNAi embryos were processed in parallel with stage-matched untreated embryos that were allowed to develop under water in the dish used for egg activation (wild-type controls), embryos that were prepared for injection under oil but not injected (alignment controls, AC), and embryos that were injected with double-stranded RNA of the extraneous gene *DsRed* (injection controls, IC). *Cal-zld* and *Cal-opa* expression levels were quantified using the RNA-seq data (see *Fold change calculations*).

### ATAC-seq library preparation and RNA isolation

The first steps were the same as in the standard ATAC-seq protocol above. Individual embryos were homogenized in cold lysis buffer, and a centrifugation step was performed to pellet the nuclei. The supernatant (~47 µl) was then removed and mixed thoroughly with 450 µl Zymo RNA lysis buffer in a fresh low-bind reaction vial. The nuclear pellet was resuspended in 7.5 µl Tagmentation buffer and the ATAC-seq protocol was continued as described above. RNA isolation proceeded immediately, or the RNA lysate was stored at −20 °C for up to ~1 week. RNA isolation was performed using the Zymo Quick-RNA Tissue/Insect Microprep Kit following the manufacturer’s instructions. Isolated RNA was for RT-qPCR and RNA-seq experiments.

### Preparation of injected embryos for ATAC-seq/ATAC-seq and RNA isolation

Following the injection, embryos were left to develop on the injection slide at 25 °C. Once the embryos had reached the desired stage, any excess oil was removed from the slide using a pipette. The embryos were then washed 6 times using deinonized water. Embryos were removed from the injection slide using a blunted tungsten needle and transferred to the lid of a low bind reaction vial. 10 μl of cold lysis buffer was added and the embryos were homogenized using a fire-sealed microcapillary tube. The ATAC-seq protocol was conducted as described above.

### ATAC-seq data processing

Demultiplexed reads were trimmed of adapters using TrimGalore! [160] (version 0.6.10, cutadapt version 4.4) and mapped to the *Clogmia albipunctata* assembly (calbi-uni4000) using Bowtie2 [161] (version 2.4.1) with option -X 2000. Suspected optical and PCR duplicates were marked by Picard MarkDuplicates (version 2.21.4) using default parameters (https://broadinstitute.github.io/picard/). Mapped, trimmed, duplicate marked reads were imported into R using the GenomicAlignments [162] (version 1.38.2) and Rsamtools [163] (version 2.18.0). Reads that mapped, were properly paired, nonsecondary and had map quality scores ≥ 10 were imported. Only reads mapping to scaffolds 1–5 and scaffold 7 were used for downstream analysis. The start and end coordinates of the reads were adjusted to account for the Tn5 interaction site. Watson strand start sites had four base pairs subtracted, and Crick-strand start sites had five base pairs subtracted [158]. Reads with mapped length ≤ 120 bp were considered to have originated from ‘open’ chromatin.

#### MACS2.

Accessible chromatin peaks from NC11 through NC14 were determined using MACS2 [97] (version 2.2.7.1) with options -f BEDPE -q 1e^−25^ on a merged dataset comprising all ATAC reads corresponding to open chromatin (length ≤ 120 bp). Peaks were assigned to a genomic feature on the basis of overlapping with one or more features of a gene (e.g., exon, intron, and promoter). Peaks that overlapped with no gene feature were classified as intergenic.

#### DESeq2.

A count matrix for differential enrichment analyses using DESeq2 [101] (version 1.42.1) was generated by counting the number of open ATAC reads (length ≤ 120 bp) that overlapped with a MAC2 identified peak. Before running DESeq2, peaks with low count values were removed. DESeq2 design parameters were specific to the experimental data set. For the WT stage-wise comparisons (e.g., NC12 versus NC11, NC13 versus NC12, NC14 versus NC13, and LNC14 versus NC14) the design parameters passed to DESeq2 were stage and sequencing platform (~instrument.atac.data + stage). For the comparison between RNAi embryos and alignment control embryos (e.g., *Cal-zld* RNAi versus alignment control and *Cal-opa* RNAi versus alignment control) the design components were embryo batch and genotype (~Experiment.Date + genotype). All control groups were given their own genotype to account for differences in embryo manipulation, and the genotype for the RNAi embryos was determined by the strength of knockdown (see Fold change calculations). The criteria for a statistical significance change were a log_2_ fold change ≥ 1 and an adjusted *p*-value ≤ 0.05 or a log_2_ fold change ≤ −1 and an adjusted *p*-value ≤ 0.05.

Dynamic peaks were defined as peaks that underwent a statistically significant change in accessibility in at least one of the WT stage wise comparisons. Different dynamic peak classes were found using the R package DEGreport [164] (R package version 1.38.5) with the parameter of the minimum group size set to 10.

#### MEME.

For S3 Fig, MEME [103] (version 5.4.1) analysis was performed using the following options -order 1 -meme-mod zoops -minw6 -psp-gen -maxw 14 -meme-nmotifs 15 -meme-searchsize 100,000 -centrimo-score 5.0 -centrimo-ethresh 10.0. For S9 Fig, we used MEME (version 5.5.8) with -order 1 -meme-mod zoops -minw4 -maxw 14 -meme-nmotifs 15 -meme-searchsize 100,000 -centrimo-score 5.0 -centrimo-ethresh 10.0. Motifs were reported from all enrichment or discovery programs within the MEME suite (i.e., MEME, STREME [104], CentriMo), unless specified otherwise. MEME was given a set of sequences containing the 100 bp sequence centered on the summit of the peak. To determine motifs enriched in wild-type ATAC-seq peaks, a set of 1,000 background sequences in genomic regions without ATAC-seq peaks was used as background sequences. To determine motifs enriched in dynamic wild-type peaks, 1,000 randomly chosen nondynamic peak sequences were provided as background sequences. To determine motifs enriched in *Cal*-*zld* sensitive or Cal-opa sensitive peaks, all peaks unaffected by *Cal*-*zld* knockdown or *Cal-opa* knockdown were provided as background sequences. Identified motifs were compared to known *Drosophila melanogaster* motifs from the following databases: onTheFly_2014, Fly Factor Survey, FLYREG, iDMMPMM, and DMMPMM databases [116,165–168].

### RNA-seq data processing

Demultiplexed reads were trimmed of adapters using TrimGalore [160] (version 0.6.10, cutadapt version 4.4; https://www.bioinformatics.babraham.ac.uk/projects/trim_galore/) and mapped to the *Clogmia albipunctata* assembly (calbi-uni4000) using Bowtie2 [161] (version 2.4.1) with default parameters and maximum fragment length (-X) of 2000. Suspected optical and PCR duplicates were marked by Picard MarkDuplicates (version 2.21.4) using default parameters. Mapped, trimmed, duplicate marked reads were imported into R using the GenomicAlignments [162] (version 1.38.2) and Rsamtools [163] (version 2.18.0). Reads that mapped, were properly paired, nonsecondary and had a map quality scores ≥ 10 were imported.

#### DESeq2.

A gene count matrix for differential enrichment analyses was generated by counting the number of RNA-seq reads that overlapped with the transcripts of a gene. Before running DESeq2, genes with low count values were removed. DESeq2 design parameters were specific to the experimental data set. For the comparison between RNAi embryos and alignment control embryos (e.g., *Cal-zld* RNAi versus alignment control and *Cal-opa* RNAi versus alignment control) the design components were embryo batch and genotype (~Experiment.Date + genotype). All control groups were given their own genotype to account for differences in embryo manipulation, and the genotype for the RNAi embryos was determined by the strength of knockdown (see Fold change calculations). The criteria for a statistically significant change were a log_2_ fold change ≥ 1 and an adjusted *p*-value ≤ 0.05 or a log_2_ fold change ≤ −1 and an adjusted *p*-value ≤ 0.05.

#### Maternal, zygotic, maternal, and zygotic classification.

Gene expression was compared in NC2/3 (maternal, ~45-min old) and syncytial stage embryos (NC11- Late NC14). We assume here that NC2/3 embryos have little to no active transcription and instead are enriched in maternally deposited mRNAs. The distribution of gene expression was plotted for both stages, and a gene was determined to be expressed if its expression level was above 2^5^ counts. Then, genes were classified based on their expression profile in NC2/3 and syncytial stage embryos. We found 227 maternal genes, 691 zygotic genes, 6,658 maternal and zygotic genes, and 7,473 genes not expressed during either stage.

#### Fold change calculations.

Fold change was calculated as the sample’s counts per million (CPM) divided by the control’s CPM for the gene of interest. First, a CPM matrix was made by counting the number of RNA-seq reads that overlapped with a gene’s transcripts and then normalizing to CPM. Fold change calculations were done with embryos grouped by experimental date (i.e., embryos from the same egg packet). For each embryo the CPM was divided by the average CPM of wild-type and alignment control embryos from the same experimental date. If a date did not have any wild-type or alignment control embryos, fold change was calculated using the average CPM of all wild-type and alignment control embryos in the data set. For *Cal-zld* RNAi experiments a fold change (FC) calculation of *Cal-zld* mRNA levels was conducted with the average of control embryos (untreated wild-type and alignment controls) from the same experimental date as reference. *Cal*-*zld* RNAi embryos with a fold change < 0.25 (knockdown efficiency greater than 75%) were selected to assess the effect of *Cal-zld* knockdown; *Cal*-*zld* RNAi embryos with a fold change ~1 or higher (no *Cal-zld* knockdown detected) and uninjected embryos (alignment controls) were selected for comparison in further analyses. Similarly, for *Cal-opa* RNAi experiments fold change calculations were done separately for each stage. The fold change calculation of *Cal-opa* mRNA levels was conducted with the average of control embryos (untreated wild-type and alignment controls) from the same experimental date as a reference. *Cal*-*opa* RNAi embryos with a fold change < 0.25 (knockdown efficiency greater than 75%) and with a fold change > 1 (no knockdown observed, 2 embryos at NC13) were used for further analysis.

### Genome assembly

We used Cantata Bio’s genome assembly services to generate a de novo reference genome for *Clogmia albipunctata* from ~70 freshly eclosed males of a 12-generation inbred line of an established laboratory culture [169]. High-fidelity (HiFi) PacBio reads were used to generate an initial draft assembly, subsequently refined with Omni-C reads (S1 Appendix). Details on sequencing and software used to assemble the genome have been described elsewhere [91]. We estimated genome heterozygosity and sequencing error rates using jellyfish to count 21-mers and GenomeScope2.0 to analyze the k-mer frequencies.

### Genome annotation

We used *RepeatModeler* [170] (version v2.0.4) to identify repeat elements in the *Clogmia albipunctata* genome and *RepeatMasker*’s [171] (version v4.1.5) *fambd.py* script to extract *Arthropoda* records from the *Dfam* database. These sequences were combined to generate a custom repeat library, which was then used to soft-mask the genome. To predict gene structures, we used RNA-seq and protein evidence. mRNA data from 26 individual embryos of various stages, pooled samples of first and fourth-instar larvae, one female and one male pupa generated in this study were mapped to the genome (S1 Table). Additionally, 11 RNA samples (9 embryonic and two adult) from other studies were downloaded from NCBI (S1 Table). For protein evidence, we downloaded all available Dipteran sequences from NCBI’s RefSeq (January 2024) and UniProt’s complete protein sequences (Release 2023_04). A detailed description of the annotation pipeline is available elsewhere [91]. Briefly, we assembled and mapped the RNA transcripts and protein sequences to the reference and used these data as evidence of gene structures and to predict gene models. *Evidence Modeler* [172] (EVM, version 2.1.0) was used to create a consensus gene set, which was further refined with Program to Assemble Spliced Alignments’ (PASA version 2.5.3) to add UTRs and identify alternative transcripts. Gene structures overlapping with repeats and transposable elements (TEs) were filtered out. Functional annotation was performed with *EggNOG-mapper* [173,174] (version 2.1.12), assigning gene names based on orthology. We assessed the final annotation quality using BUSCO (Benchmarking Universal Single-Copy Orthologs) [175,176] on the predicted protein sequences. Finally, we analyzed synteny between the *C. albipunctata* and *D. melanogaster* genomes using *MCScanX* [177] (primary release) with parameters: match_score = 50 (default), match size = 20 (minimum genes per syntenic block), gap_penalty = −1 (default), and max_gaps = 100. Results were visualized using the *circlize* R package [178] (version 4.1.2). Annotations of *Cal-eve1, Cal-eve2, Cal-tll1,* and *Cal-tll2* were manually corrected in the final output file (.gff3) based on RNA-seq data and transcript data available on NCBI.

### TF identification

Any gene in our annotation of the Clogmia genome that contained the Cluster-of-Orthologous-Gene (COG) symbol “K” for transcription or the Gene Ontology (GO) term “0003700” for DNA-binding TF activity was treated as a putative TF gene.

### Genome browser

The genomic data produced in this study can be further interrogated using a genome browser ecosystem centered on JBrowse2 [179] and delivered as a cloud image designed for individual use, currently available on NSF’s Jetstream2 cloud platform, with a portable Docker image with documentation and BSGenome package created for R. Jetstream2’s user-friendly interface (image: *Clogmia albipunctata* Genome Resources) [180,181] integrates tools for genomic analysis, including BLAST search (SequenceServer2.0) [182], CRISPR guide RNA design (modified crisprDesigner) [183], R shiny-driven differential gene expression (DGE) analysis (freecount) [184], and synteny mapping for comparative genomics (ShinySyn) [185]. Each tool is connected back to the browser, leveraging JBrowse2’s ability to dynamically visualize and contextualize the genomic data. BLAST results link directly to genomic regions, enabling users to explore expression tracks, variants, and synteny relationships. CRISPR tools provide sgRNA design with metrics for selection and visualization of target regions alongside expression data, off-target effects, and variants. DGE analysis visualizations are linked back to the genome browser for further exploration. All gene model annotations in the browser connect to common external databases, such as NCBI, Uniprot, FlyBase, and Google Scholar, for further exploring and interpreting the data. Future enhancements will include expanded sgRNA profiling, and further optimization of workflows, ensuring this ecosystem remains a powerful and accessible solution for the use of our genomic data presented here and elsewhere [91] for research and education. Installation instructions for accessing the *Clogmia albipunctata* genome browser and related tools are available at https://github.com/kallistaconsulting/genomic_resources_clogmia. The container requires a Linux web server on any cloud platform. The docker was developed on Jetstream2, which can be accessed via instructions at https://jetstream-cloud.org/get-started/index.html. This on-demand container-based model reduces resource costs and security concerns compared to hosting a centralized site. Administrative tools (e.g., automated reboot scripts, system service management, load balancing for multi-user groups or workshops) enhance usability while minimizing operational overhead.

## Supporting information

S1 Table Relating to Fig 1 and Table 2: RNA-seq samples used for annotation. (XLSX)

S2 Table Relating to Fig 1: Homologs of early segmentation genes in *Clogmia albipunctata.* Homologs were identified by reciprocal BLASTp [186] searches between *Drosophila melanogaster* and *Clogmia albipunctata*. Note that *knirps* and *knirps-like* as well as *sloppy-paired 1*, *sloppy-paired 2*, and *forkhead domain 19B* resulted from gene duplications specific to the Drosophila lineage, while *Cal-knrl1* and *Cal-knrl2* as well as *Cal-slp1*, *Cal-slp2*, and *Cal-slp3* resulted from gene duplication specific to the Clogmia lineage.
(XLSX)

S3 Table *Cal*-*zld* RT-qPCR data. Fold change calculated using the ∆∆Ct method. Reference gene *Cal*-*rpl35*. Target gene *Cal*-*zld*. Percent knockdown was calculated as 100−(FC*100).
(XLSX)

S4 Table *Cal*-*opa* RT-qPCR data. Fold change calculated using the ∆∆Ct method. Reference gene *Cal*-*rpl35*. Target gene *Cal*-*opa*. Percent knockdown was calculated as 100−(FC*100). Indicated is whether the RNA was isolated immediately or in parallel with an ATAC-seq library preparation. RNA was either isolated using the Zymo Quick-RNA Tissue/Insect Microprep Kit following the manufacturer’s instructions kit or following an ATAC-seq library preparation using the protocol by Li and colleagues [187]. Briefly following the tagmentation reaction, mRNA was isolated from the supernatant using Dynabeads Oligo (dT)25 beads. Subsequent washes were performed and mRNA was eluted directly from the beads.
(XLSX)

S1 Fig Relating to Fig 2: genomic distribution of ATAC-seq peaks at nuclear cycles NC11, NC12, NC13, NC14, and Late NC14. (TIF)

S2 Fig Relating to Fig 2: dynamic ATAC-seq peak groups. Dynamic peak groups were generated using DEG reports [164] (R package version 1.38.5). Y-axis, Z-score of abundance. X-axis, developmental stage. Colored bars mark manually established super-groups with similar features described as follows: incrementally opens (yellow bar; Groups 8, 12, 20); incrementally opens until NC13 (red bar; Group 10); opens after NC13 (green; Groups 15, 14, 4); incrementally opens until NC14, then closes (blue; Group 6); incrementally opens until NC13, then closes (red-brown; Groups 3, 1, 7, 18); incrementally closes (violet; Groups 5, 13); closes after NC14 (orange; Group 11); other (olive; Groups 9, 17, 16, 21, 19, 15).
(TIF)

S3 Fig Relating to Fig 2: enriched motifs in ATAC-seq peaks. Enriched motifs identified by MEME for 8 sets of peaks: NC11 peaks (*n* = 5,013), NC12 peaks (*n* = 5,926), NC13 peaks (*n* = 7,586), NC14 peaks (*n* = 16,147), Late NC14 peaks (*n* = 11,628), all peaks (*n* = 32,157), dynamic peaks (*n* = 6,930), and nondynamic peaks (right column; *n* = 25,227). The top five motifs discovered by MEME are reported for each group. Motifs are shown as PWM logos with significance (*E*-value). DNA binding proteins of *Drosophila melanogaster* that bind to these or similar motifs as identified by MEME are indicated. We note that although not identified by MEME, CLAMP has been shown to bind GA-rich motifs and Cg has been shown to bind (CA)n like motifs [105–108].
(TIF)

S4 Fig Protein alignment of conserved Zelda domains. Cal-Zld sequence is shown above Zld sequences of *Tribolium castaneum* (Tc-Zld, XP_001812268.1) and *Drosophila melanogaster* (Dme-Zld, NP_608356.1). Alignment was performed using MAFFT [188] (v7) with the default settings. Amino acid color indicates physio-chemical property (based on Zappo Color Scheme). Important protein domains are indicated [110].
(TIF)

S5 Fig Time-course of *Cal*-*zld* expression. Gene model in pale yellow. RNA-seq data in counts per million (CPM). Y-axis for NC2/3 (maternal, ~45-min old embryos) and NC11: 0–10 CPM. Y-axis for NC12 and NC13: 0–25 CPM. Y-axis for NC14 and Late NC14 (LCN14): 0–120 CPM.
(TIF)

S6 Fig Relating to Figs 3 and 4: identification of Cal-zld RNAi embryos with strong knockdowns. Plot of *Cal-zld* fold change for individual embryos. Each embryo is represented by a circle. Embryos are grouped along the x-axis as indicated; spacing within a group was done arbitrarily for visualization. The treatment group of each embryo is indicated by color. Fold change for each embryo was calculated by dividing the embryo’s CPM of *Cal-zld* by the average of wild-type and alignment control *Cal-zld* CPMs. Fold change was calculated using embryos from a single female (same activation batch). If controls from the same batch were lost, fold change was calculated using the average *Cal-zld* CPM of all wild-type and alignment control embryos in the data set. *Cal*-*zld* RNAi embryos with fold change < 0.25 were selected for the zldKD group (*n* = 4). Alignment control embryos (AC; *n* = 5) and *Cal*-*zld* RNAi embryos with fold change ~1 or higher (zldnoKD; *n* = 3) served as controls. A *Cal*-*zld* RNAi embryos with intermediate *Cal-zld* fold change (intKD) was excluded from further analysis. Wt, wild-type embryos.
(TIF)

S7 Fig Relating to Fig 4: comparison of downregulated and upregulated ATAC-seq peaks in response to *Cal-zld* RNAi. **A)** Overlap of downregulated peaks between zldKD-vs-AC and zldKD-vs-zldnoKD, and between zldKD-vs-zldnoKD and zldnoKD -vs-AC. **B)** Overlap of upregulated peaks between zldKD-vs-AC and zldKD-vs-zldnoKD, and between zldKD-vs-zldnoKD and zldnoKD -vs-AC.
(TIF)

S8 Fig Relating to Fig 4: *Cal-zld* sensitive ATAC-seq peaks in dynamic and nondynamic groups. Peaks in different groups are ordered by *Cal-zld* sensitivity. Peaks that gained accessibility are shown in blue and peaks that lost accessibility are shown in red. The number of peaks and percentage relative to all down- or upregulated peaks are indicated.
(PDF)

S9 Fig Relating to Fig 4: MEME analysis of *Cal-zld*-sensitive ATAC-seq peaks. Top three differentially enriched motifs (within 50 bp of the peak summit) of downregulated or upregulated ATAC-seq peaks relative to all consistently nonsensitive peaks are shown for zldKD-vs-AC, zldKD-vs-zldnoKD, and the negative control, zldnoKD-vs-AC. Motifs for each comparison are shown as a position weight matrix logo with decreasing significance (increasing *E*-value) from left to right. DNA binding proteins of *Drosophila melanogaster* that bind to these or similar motifs as identified by MEME are indicated. We note that although not identified by MEME, CLAMP has been shown to bind GA-rich motifs [105–107].
(PDF)

S10 Fig Relating to Figs 5, 6, 7, and 8: identification of Cal-opa RNAi embryos with strong knockdowns. Plots of *Cal-opa* fold change for individual embryos at NC12 and NC13. Each embryo is represented by a circle. Embryos are grouped along the x-axis as indicated; spacing within a group was done arbitrarily for visualization. The treatment group of each embryo is indicated by color. Fold change for each embryo was calculated by dividing the embryo’s CPM of *Cal-opa* by the average of wild-type and alignment control *Cal-opa* CPMs. Fold change was calculated using embryos from a single female (same activation batch). *Cal*-*opa* RNAi embryos with fold change < 0.25 were selected for the opaKD group (*n* = 4 for each stage). *Cal*-*opa* RNAi embryos with intermediate *Cal-opa* fold change (intKD) and one NC12 embryo with *Cal-opa* fold change > 1 (noKD) were excluded from further analysis. Wt, wild-type embryos.
(TIF)

S11 Fig Relating to Figs 5 and 6: comparisons of opanoKD-vs-AC RNA-seq and ATAC-seq peaks at NC13. A) Downregulated (red) and upregulated (blue) RNA-seq peaks (no TF genes; cf. S6 Appendix). B) Downregulated (red) and upregulated (blue) ATAC-seq peaks (cf. S7 Appendix).
(TIF)

S12 Fig Relating to Figs 5 and 6: RNA-seq and ATAC-seq tracks of *Cal-hb.* RNA-seq and ATAC-seq tracks are shown for NC12 and NC13 with alignment control in blue, injection control in light blue, *Cal-opa* RNAi without KD in green, and *Cal-opa* RNAi with KD in red. Gene models are shown in pale yellow. ATAC-seq peaks with significant changes in accessibility are highlighted in pink (cf. S7 Appendix). The scale of the y-axis (CPM) is adjusted for each data type but kept constant between stages and is indicated with numbers in square brackets: [0–20] for RNA-seq data; [0–25] for ATAC-seq data.
(TIF)

S13 Fig Maximum-likelihood tree of Sloppy-paired homologs. Reciprocal protein BLAST was performed using Slp1 (NP_476730.1), Slp2 (NP_476834.1), Fd19B (NP_608369.1), and Crocodile (Croc; outgroup; NP_524202.1) as queries. Homologous protein sequences in *Nematostella vectensis*, *Mus musculus*, *Caenorhapditis elegans*, *Ischnura elegans*, *Gryllus longicernus*, *Nasonia vitripennis*, *Tribolium castaneum*, *Bombyx mori*, *Clunio marinus*, *Anopheles gambiae*, *Lutzomyia longipalpis*, *Bradysia coprophila*, *Hermetia illucens*, *Condylostylus longicornis*, and *Episyrphus balteatus* were identified by reciprocal Protein BLAST (https://blast.ncbi.nlm.nih.gov/Blast.cgi) [186]. Homologous sequences in *Limnephilus lunatus* [189], *Panorpa germanica* [190], and *Nephrotoma appendiculata* [191] were identified by reciprocal BLAST in oHoGeneious Prime 2024.0.7 (https://www.geneious.com/), using respective transcript and protein fasta files available at ensemble-Darwin Tree of life (https://projects.ensembl.org/darwin-tree-of-life/). *E* value cutoff threshold was set to 0.05 and maximum number of target sequences set to 100. Identified sequences (for accession numbers see tree) were aligned using MAFFT v.7.526 with the L-INS-i strategy (https://mafft.cbrc.jp/alignment/software/) [188]. Maximum-likelihood trees were constructed using IQ-TREE v.2.3.6. [121]. The best molecular substitution model for each partition under the Bayesian information criterion was selected by partition merging strategy with ModelFinder [122]. Maximum-likelihood trees were constructed under the selected substitution models (VT + F + I + G4), with branch support values estimated by the ultrafast bootstrap approximation [121] using 1,000 replicates. A majority-rule consensus tree was then generated based on the bootstrap results. The consensus tree was visualized on FigTree v.1.4.4 (http://tree.bio.ed.ac.uk/software/figtree/) [192]. Nematostella Croc sequence was manually chosen as root.
(PDF)

S14 Fig The evolution of *sloppy-paired.* Homologs of *sloppy-paired* are mapped onto simplified trees of Metazoa (top) and Diptera (bottom). Orthologs of *slp1* (green), *Fd19B* (orange), *slp2* (blue), and the inferred progenitors *slp1/fd19B/slp2* (joined rectangles in green, orange, and blue) and *Slp1/fd19B* (joined rectangles in green and orange) are color-coded. Inferred gene duplications are indicated by white spaces between colored boxes.
(TIF)

S1 Movie Time-lapse movie from ~NC11 to gastrulation. Embryos from left to right; 1) *Cal*-*zld* RNAi, 2) *Cal*-*zld* RNAi, 3) *Cal*-*zld* RNAi, 4) alignment control, and 5) *Cal*-*zld* RNAi. Transmitted light imaging was performed on a Leica DM5000B at 1-min intervals. Imaging was stopped and needed to be restarted twice, this accrues at the ~30 s and ~65 s marks. Scale bar 100 µM.
(MOV)

S2 Movie Loss of *Drosophila melanogaster zelda* impairs cellularization and cytoplasmic clearing during zygotic genome activation. This movie is a composite of two independently recorded movies aligned to coincide with the beginning of nuclear cycle 14, both of Cry2-zelda embryos. Cry2-zelda embryos express a chimeric Zld protein fused to the light-inducible Cry2 module, allowing *zelda* loss of function upon exposure to blue light [56]. The top embryo was imaged at a permissive wavelength (zld+, 594 nm) and the bottom one was imaged at the restrictive wavelength (zld−, 488 nm) on a confocal microscope with a 30-s frame rate. The timestamp indicates minutes relative to the beginning of NC14. Note that while the zld+ embryo develops clear cortical cytoplasm and cellularizes during the ~1h period of NC14, the zld- embryo retains dark cytoplasm at the basal surface of the nuclei and fails to undergo cellularization, prior to loss of epithelial integrity (~ +45 min, lower right). Scale bar, 50 µm.
(MP4)

S1 Data Relating to Fig 2: ATAC-seq count matrix (sheet 1) and metadata (sheet 2) for time course of wild-type embryos. (XLSX)

S2 Data Relating to Fig 3: RNA-seq count matrices and metadata for time course of wild-type embryos (sheet 1 and 2) and *Cal-zld* RNAi embryos (sheet 3 and 4). (XLSX)

S3 Data Relating to Fig 4: ATAC-seq count matrix (sheet 1) and metadata (sheet 2) for *Cal-zld* RNAi and control embryos. (XLSX)

S4 Data Relating to Fig 5: RNA-seq count matrices and metadata for *Cal-opa* RNAi and control embryos at NC12 (sheet 1 and 2) and NC13 (sheet 3 and 4). (XLSX)

S5 Data Relating to Fig 6: ATAC-seq count matrices and metadata for *Cal-opa* RNAi and control embryos at NC12 (sheet 1 and 2) and NC13 (sheet 3 and 4). (XLSX)

S1 Appendix Relating to Fig 1: Dovetail/Cantata Bio Hifiasm and HiRise scaffolding reports. (PDF)

S2 Appendix Relating to Fig 1: blast hits of annotated *Clogmia albipunctata* genes with flybaseID. (XLSX)

S3 Appendix Relating to Fig 2: chromatin accessibility at stages NC11, NC12, NC13, NC14, and Late NC14. (XLSX)

S4 Appendix Relating to Fig 3: *Cal-zld* RNAi RNA-seq analysis. 1) zldKD versus alignment control. 2) zldKD versus zldnoKD. 3) zldnoKD versus alignment control.
(XLSX)

S5 Appendix Relating to Fig 4: *Cal-zld* RNAi ATAC-seq analysis. 1) zldKD versus alignment control. 2) zldKD versus zldnoKD. 3) zldnoKD versus alignment control.
(XLSX)

S6 Appendix Relating to Fig 5: *Cal-opa* RNAi RNA-seq analysis. 1) NC12 opaKD versus alignment control. 2) NC12 opaKD versus injection control. 3) NC13 opaKD versus alignment control. 4) NC13 opaKD versus alignment control. 5) NC13 opaKD versus opanoKD. 6) NC13 opanoKD versus alignment control.
(XLSX)

S7 Appendix Relating to Fig 6: *Cal-opa* RNAi ATAC-seq analysis. 1) NC12 opaKD versus alignment control. 2) NC12 opaKD versus injection control. 3) NC13 opaKD versus alignment control. 4) NC13 opaKD versus alignment control. 5) NC13 opaKD versus opanoKD. 6) NC13 opanoKD versus alignment control.
(XLSX)

## Abbreviations

AP: anterior-posterior
BUSCO: Benchmarking Universal Single-Copy Orthologs
COG: Cluster-of-Orthologous-Gene
CPM: counts per million
DGE: differential gene expression
FC: fold change
GO: Gene Ontology
HCR: hybridization chain reaction
NC: nuclear cycle
TEs: transposable elements
TF: transcription factor
TSS: transcription start site
UDI: Unique Dual Indexes
ZGA: zygotic genome activation.
