# Supplementary material for: Asymmetric chromatin accessibility underlies anterior-posterior axis specification in moth fly embryos
Source: PLoS Biol. 2026 Aug 6;24(8):e3003896. doi: 10.1371/journal.pbio.3003896 (PMC13446688; doi:10.1371/journal.pbio.3003896)

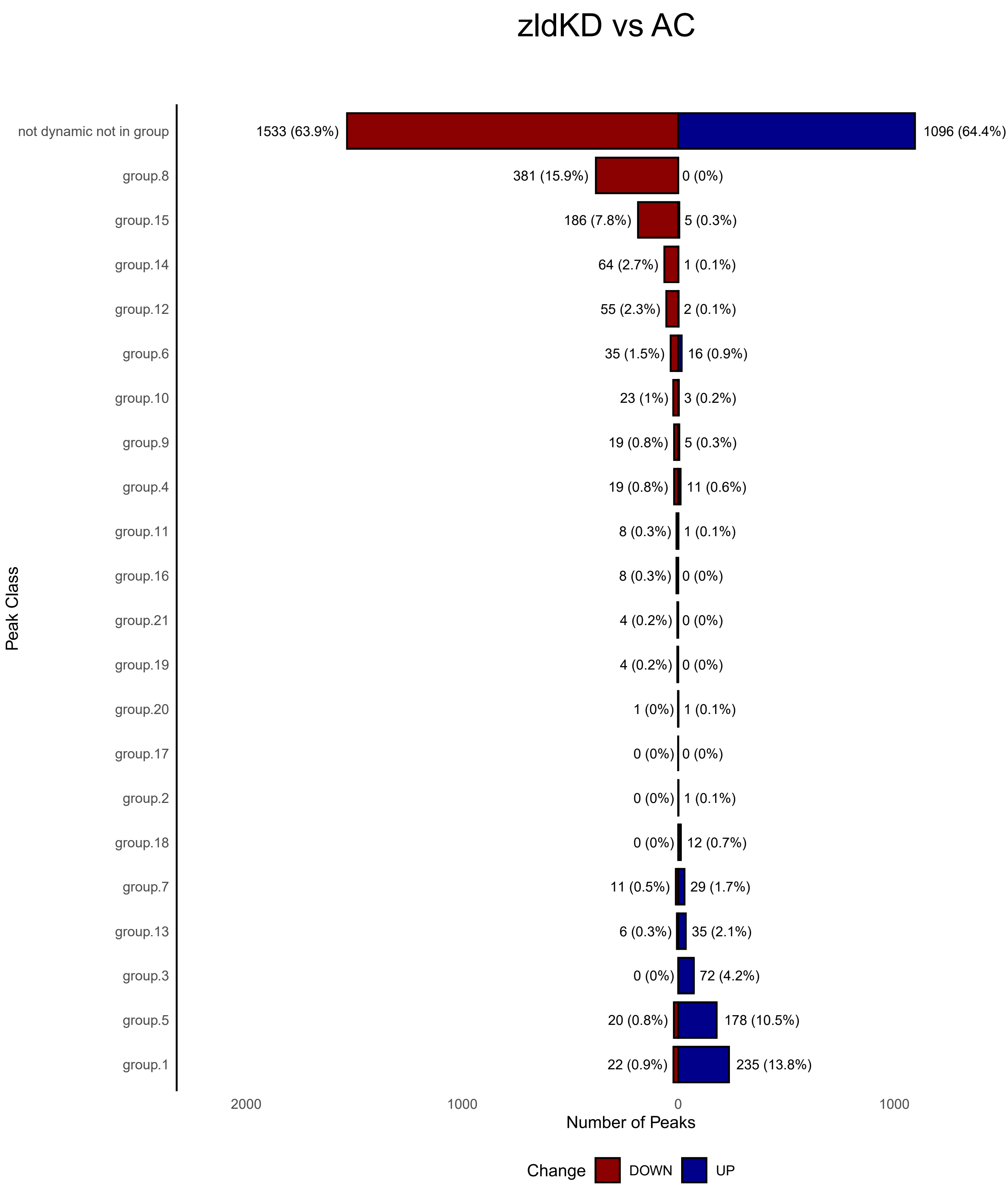
zldKD vs AC
Peak Class
not dynamic not in group
group.8
group.15
group.14
group.12
group.6
group.10
group.9
group.4
group.11
group.16
group.21
group.19
group.20
group.17
group.2
group.18
group.7
group.13
group.3
group.5
group.1
1533 (63.9%)
1096 (64.4%)
381 (15.9%)
0 (0%)
186 (7.8%)
5 (0.3%)
64 (2.7%)
1 (0.1%)
55 (2.3%)
2 (0.1%)
35 (1.5%)
16 (0.9%)
23 (1%)
3 (0.2%)
19 (0.8%)
5 (0.3%)
19 (0.8%)
11 (0.6%)
8 (0.3%)
1 (0.1%)
8 (0.3%)
0 (0%)
4 (0.2%)
0 (0%)
4 (0.2%)
0 (0%)
1 (0%)
1 (0.1%)
0 (0%)
0 (0%)
0 (0%)
1 (0.1%)
0 (0%)
12 (0.7%)
11 (0.5%)
29 (1.7%)
6 (0.3%)
35 (2.1%)
0 (0%)
72 (4.2%)
20 (0.8%)
178 (10.5%)
22 (0.9%)
235 (13.8%)
2000
1000
0
1000
Number of Peaks
Change
DOWN
UP

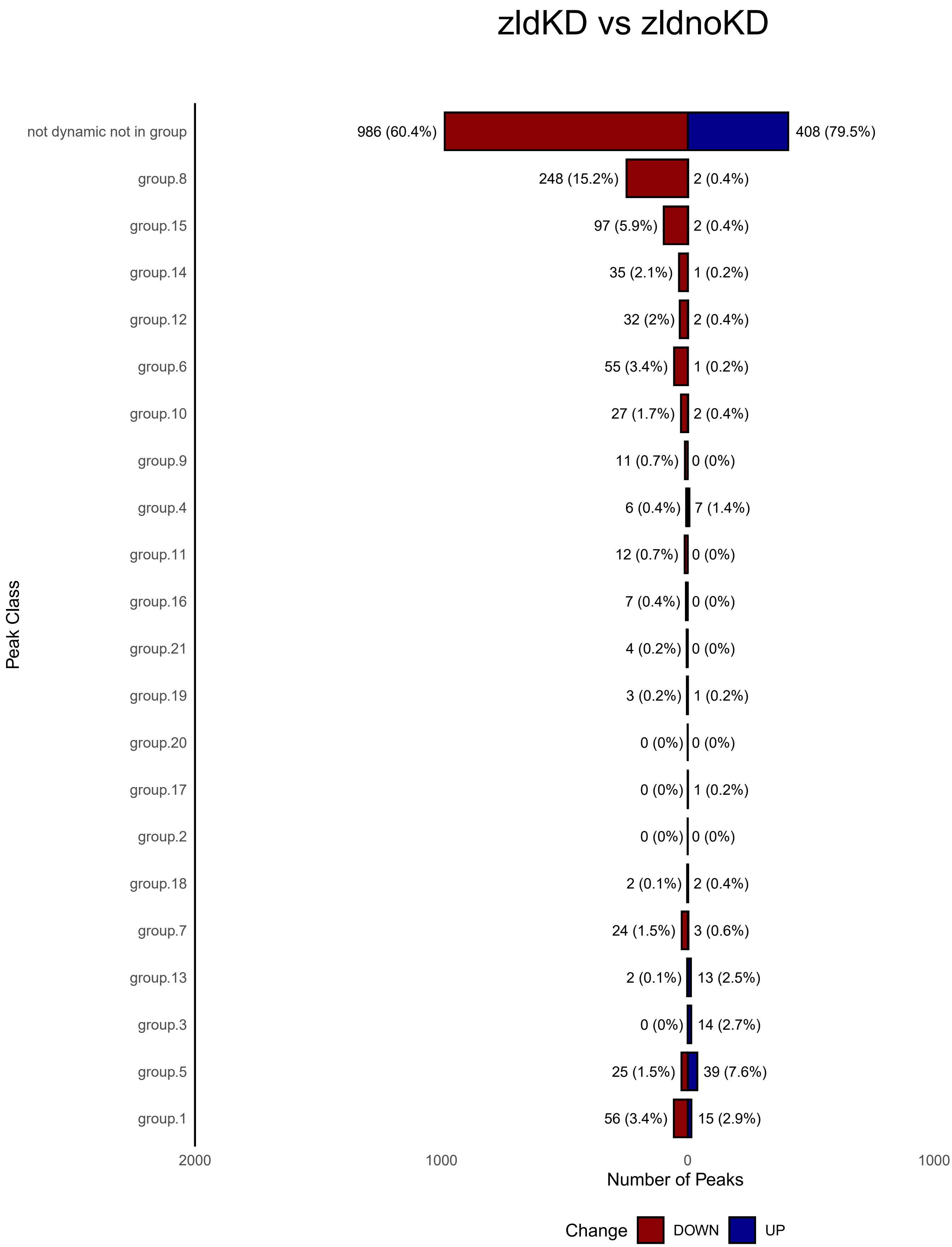

zldKD vs zldnoKD
Peak Class
not dynamic not in group
group.8
group.15
group.14
group.12
group.6
group.10
group.9
group.4
group.11
group.16
group.21
group.19
group.20
group.17
group.2
group.18
group.7
group.13
group.3
group.5
group.1
986 (60.4%)
408 (79.5%)
248 (15.2%)
2 (0.4%)
97 (5.9%)
2 (0.4%)
35 (2.1%)
1 (0.2%)
32 (2%)
2 (0.4%)
55 (3.4%)
1 (0.2%)
27 (1.7%)
2 (0.4%)
11 (0.7%)
0 (0%)
6 (0.4%)
7 (1.4%)
12 (0.7%)
0 (0%)
7 (0.4%)
0 (0%)
4 (0.2%)
0 (0%)
3 (0.2%)
1 (0.2%)
0 (0%)
0 (0%)
0 (0%)
1 (0.2%)
0 (0%)
0 (0%)
2 (0.1%)
2 (0.4%)
24 (1.5%)
3 (0.6%)
2 (0.1%)
13 (2.5%)
0 (0%)
14 (2.7%)
25 (1.5%)
39 (7.6%)
56 (3.4%)
15 (2.9%)
2000
1000
0
1000
Number of Peaks
Change
DOWN
UP

zldnoKD vs AC

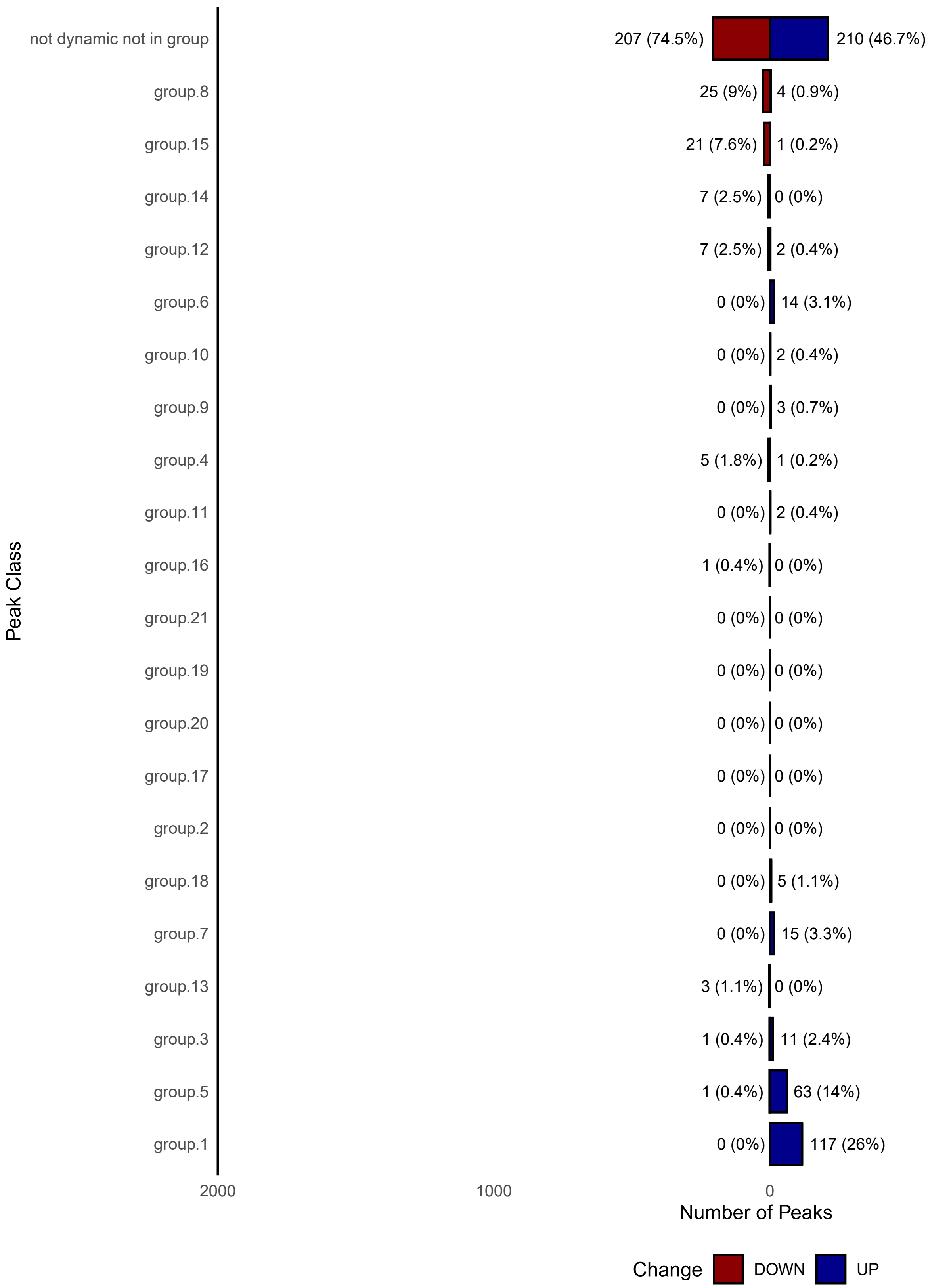

Supplement: Supplementary file 1 [file pbio.3003896.s012.pdf]
