# Supplementary material for: Asymmetric chromatin accessibility underlies anterior-posterior axis specification in moth fly embryos
Source: PLoS Biol. 2026 Aug 6;24(8):e3003896. doi: 10.1371/journal.pbio.3003896 (PMC13446688; doi:10.1371/journal.pbio.3003896)

zldKD ATAC-seq MEME Analysis Differential Enrichment mode

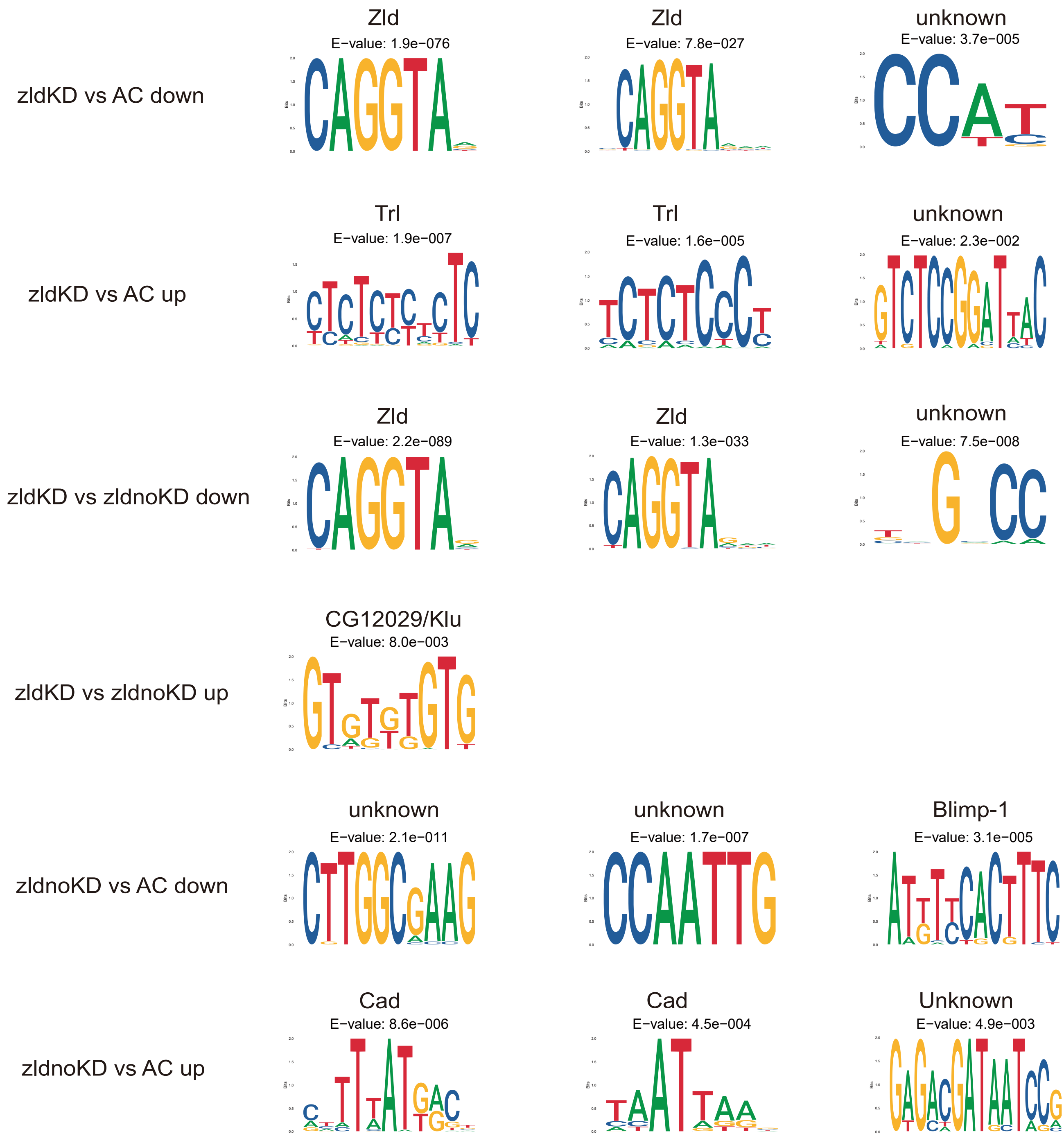

Supplement: Supplementary file 2 [file pbio.3003896.s013.pdf]
