# Supplementary material for: Asymmetric chromatin accessibility underlies anterior-posterior axis specification in moth fly embryos
Source: PLoS Biol. 2026 Aug 6;24(8):e3003896. doi: 10.1371/journal.pbio.3003896 (PMC13446688; doi:10.1371/journal.pbio.3003896)
Supplement: Supplementary file 3 [file pbio.3003896.s017.pdf]

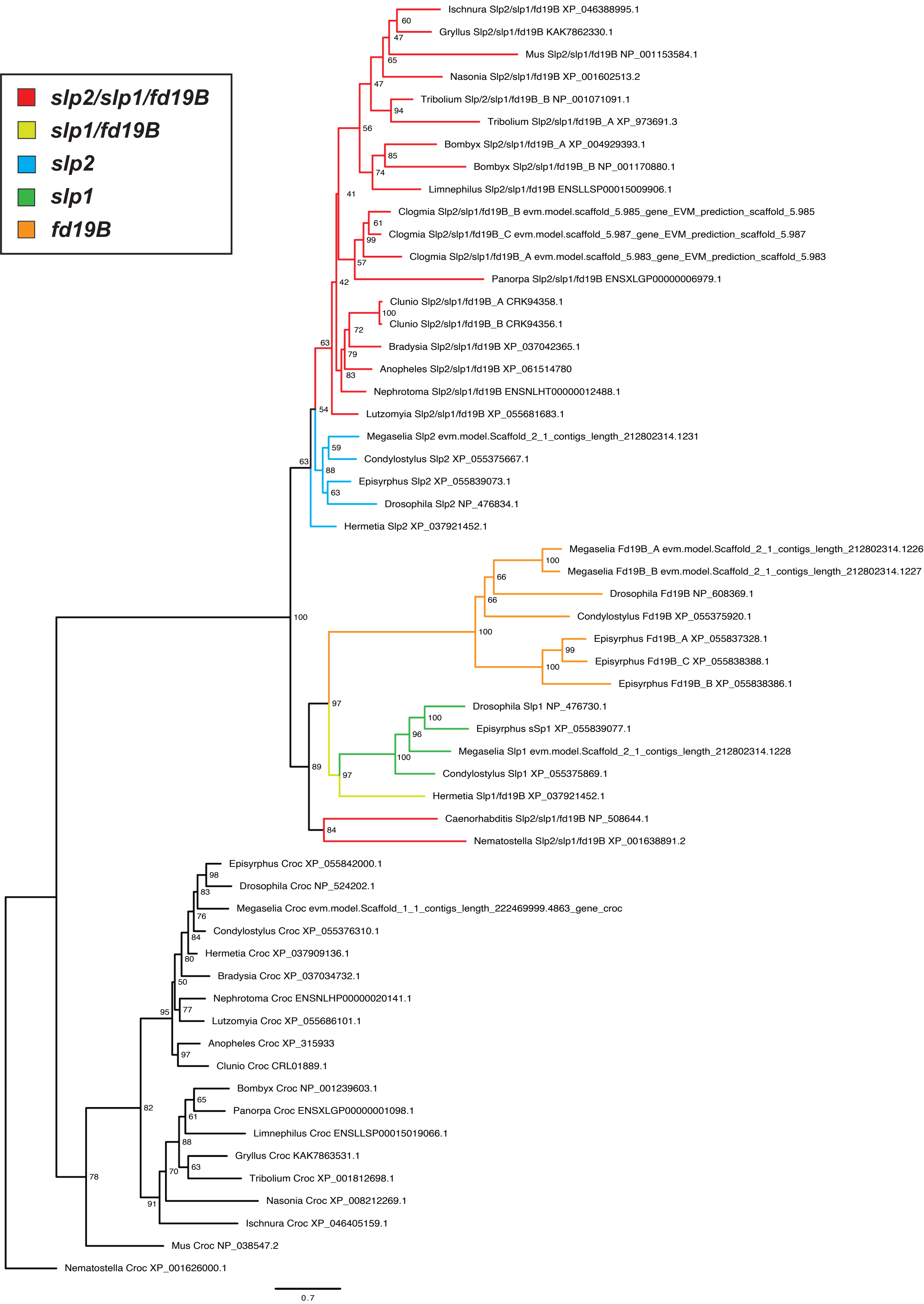

slp2/slp1/fd19B
slp1/fd19B
slp2
slp1
fd19B
Ischnura Slp2/slp1/fd19B XP_046388995.1
Gryllus Slp2/slp1/fd19B KAK7862330.1
Mus Slp2/slp1/fd19B NP_001153584.1
Nasonia Slp2/slp1/fd19B XP_001602513.2
Tribolium Slp/2/slp1/fd19B_B NP_001071091.1
Tribolium Slp2/slp1/fd19B_A XP_973691.3
Bombyx Slp2/slp1/fd19B_A XP_004929393.1
Bombyx Slp2/slp1/fd19B_B NP_001170880.1
Limnephilus Slp2/slp1/fd19B ENSLLSP00015009906.1
Clogmia Slp2/slp1/fd19B_B evm.model.scaffold_5.985_gene_EVM_prediction_scaffold_5.985
Clogmia Slp2/slp1/fd19B_C evm.model.scaffold_5.987_gene_EVM_prediction_scaffold_5.987
Clogmia Slp2/slp1/fd19B_A evm.model.scaffold_5.983_gene_EVM_prediction_scaffold_5.983
Panorpa Slp2/slp1/fd19B ENSXLGP00000006979.1
Clunio Slp2/slp1/fd19B_A CRK94358.1
Clunio Slp2/slp1/fd19B_B CRK94356.1
Bradysia Slp2/slp1/fd19B XP_037042365.1
Anopheles Slp2/slp1/fd19B XP_061514780
Nephrotoma Slp2/slp1/fd19B ENSNLHT00000012488.1
Lutzomyia Slp2/slp1/fd19B XP_055681683.1
Megaselia Slp2 evm.model.Scaffold_2_1_contigs_length_212802314.1231
Condylostylus Slp2 XP_055375667.1
Episyrphus Slp2 XP_055839073.1
Drosophila Slp2 NP_476834.1
Hermetia Slp2 XP_037921452.1
Megaselia Fd19B_A evm.model.Scaffold_2_1_contigs_length_212802314.1226
Megaselia Fd19B_B evm.model.Scaffold_2_1_contigs_length_212802314.1227
Drosophila Fd19B NP_608369.1
Condylostylus Fd19B XP_055375920.1
Episyrphus Fd19B_A XP_055837328.1
Episyrphus Fd19B_C XP_055838388.1
Episyrphus Fd19B_B XP_055838386.1
Drosophila Slp1 NP_476730.1
Episyrphus sSp1 XP_055839077.1
Megaselia Slp1 evm.model.Scaffold_2_1_contigs_length_212802314.1228
Condylostylus Slp1 XP_055375869.1
Hermetia Slp1/fd19B XP_037921452.1
Caenorhabditis Slp2/slp1/fd19B NP_508644.1
Nematostella Slp2/slp1/fd19B XP_001638891.2
Episyrphus Croc XP_055842000.1
Drosophila Croc NP_524202.1
Megaselia Croc evm.model.Scaffold_1_1_contigs_length_222469999.4863_gene_croc
Condylostylus Croc XP_055376310.1
Hermetia Croc XP_037909136.1
Bradysia Croc XP_037034732.1
Nephrotoma Croc ENSNLHP00000020141.1
Lutzomyia Croc XP_055686101.1
Anopheles Croc XP_315933
Clunio Croc CRL01889.1
Bombyx Croc NP_001239603.1
Panorpa Croc ENSXLGP00000001098.1
Limnephilus Croc ENSLLSP00015019066.1
Gryllus Croc KAK7863531.1
Tribolium Croc XP_001812698.1
Nasonia Croc XP_008212269.1
Ischnura Croc XP_046405159.1
Mus Croc NP_038547.2
Nematostella Croc XP_001626000.1
0.7
