# Supplementary material for: Asymmetric chromatin accessibility underlies anterior-posterior axis specification in moth fly embryos
Source: PLoS Biol. 2026 Aug 6;24(8):e3003896. doi: 10.1371/journal.pbio.3003896 (PMC13446688; doi:10.1371/journal.pbio.3003896)
Supplement: Supplementary file 4 [file pbio.3003896.s026.pdf]

UNI4000 - Hifiasm Report

June 16, 2023
Clogmia albipunctata
Urs Schmidt-Ott
University of Chicago

Contents

- Input Summary
- Overview
- BUSCO
- Blobplot Image
- Materials and Methods
- References
- Software Versions

Input Data

LIMS ID	Number of Reads	Bp (Gb)	Genome Size Estimate	Coverage
UNI4000	2,410,131	36.3	0.3	121x

Overview

Assembly	Total Length (bp)	N50	L50	N90	L90
Hifiasm Assembly	321,236,086	48,516,489	4	8,354,291	8
Primary Filtered Assembly	293,310,986	50,360,478	3	29,329,666	6

BUSCO

Assembly	Complete BUSCOs (C)	Complete and single-copy BUSCOs (S)	Complete and duplicated BUSCOs (D)	Fragmented BUSCOs (F)	Missing BUSCOs (M)	Total BUSCO groups searched
Hifiasm Assembly	250 (98.04%)	249 (97.65%)	1	2	3	255
Primary Filtered Assembly	245 (96.08%)	244 (95.69%)	1	2	8	255

- BUSCO version is: 4.0.5
- The lineage dataset is: eukaryota_odb10 (Creation date: 2020-09-10, number of species: 70, number of BUSCOs: 255)

Blobplot Image

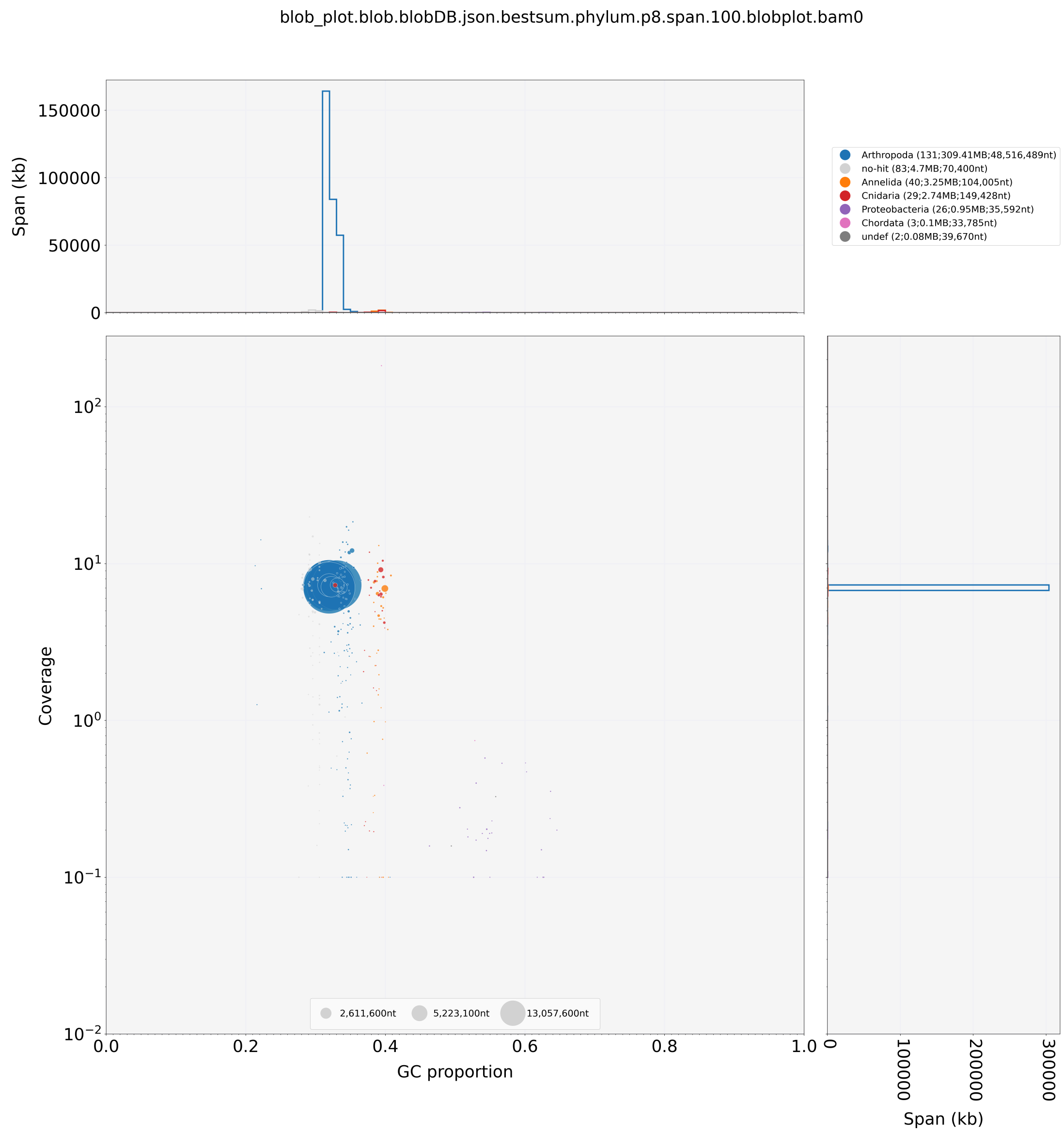

Materials and Methods

36.3 gigabase-pairs of PacBio CCS reads were used as an input to Hifiasm[1] v0.15.4-r347 with default parameters.

Blast results of the Hifiasm output assembly (`hifiasm.p_ctg.fa`) against the nt database were used as input for blobtools[2] v1.1.1 and scaffolds identified as possible contamination were removed from the assembly (`filtered.asm.cns.fa`). Finally, purge_dups[3] v1.2.5 was used to remove haplotigs and contig overlaps (`purged.fa`).

For additional software version information, please see our conda environment below.

References

1. Cheng, H., Concepcion, G.T., Feng, X., Zhang, H., Li H. Haplotype-resolved de novo assembly using phased assembly graphs with hifiasm. Nat Methods 18, 170-175 (2021). https://doi.org/10.1038/s41592-020-01056-5

2. Laetsch DR, and Blaxter ML. BlobTools: Interrogation of genome assemblies [version 1; peer review: 2 approved with reservations]. F1000Research 2017, 6:1287 https://doi.org/10.12688/f1000research.12232.1

3. Guan D, McCarthy SA, Wood J, Howe K, Wang Y, Durbin R. Identifying and removing haplotypic duplication in primary genome assemblies. Bioinformatics. 2020 May 1;36(9):2896-2898. doi: 10.1093/bioinformatics/btaa025. PMID: 31971576; PMCID: PMC7203741.

Software Versions

Package	Version
awscli	1.20.0
bioawk	1.0
blas	1.0
blast	2.13.0
blobtools	1.1.1
minimap2	2.21
numpy	1.19.1
pandas	1.1.3
pip	20.2.4
pyqt	5.9.2
pysam	0.15.4
python	3.7.6
qt	5.9.7
samtools	1.9
wtdbg	2.5

HiRise Scaffolding Report

August 04, 2023
Clogmia albipunctata
Urs SchmidtOtt
University of Chicago

Contents

- Overview
- Contiguity Metrics
- BUSCO
- Pair Size Distribution
- Scaffolding Summary
- Materials & Methods

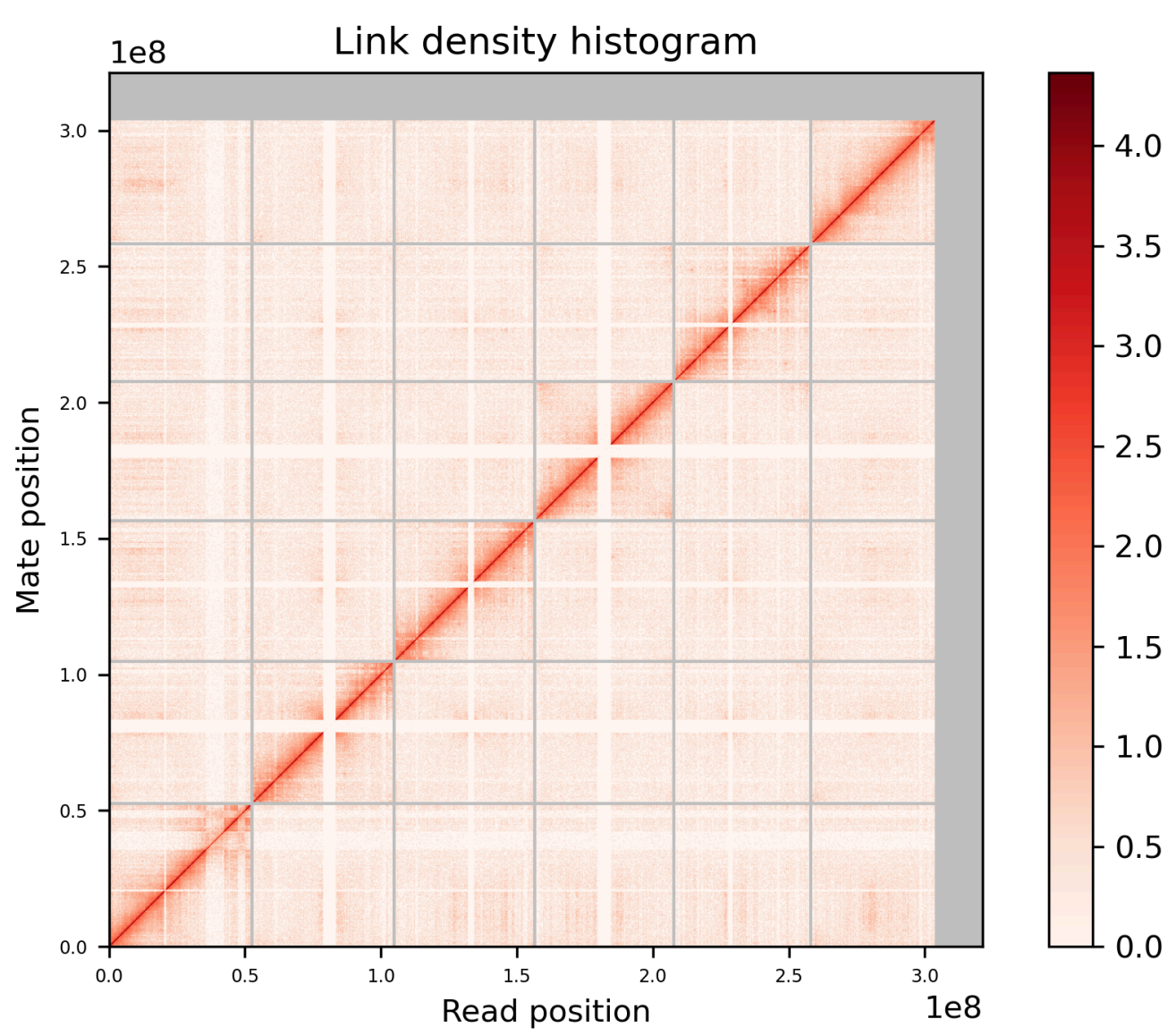

Overview

Assembly	Total Length (bp)	N50	L50	N90	L90
Input Assembly	321,236,086	48,516,489	4	8,354,291	8
Dovetail HiRise Assembly	321,236,686	51,218,107	4	46,132,707	6

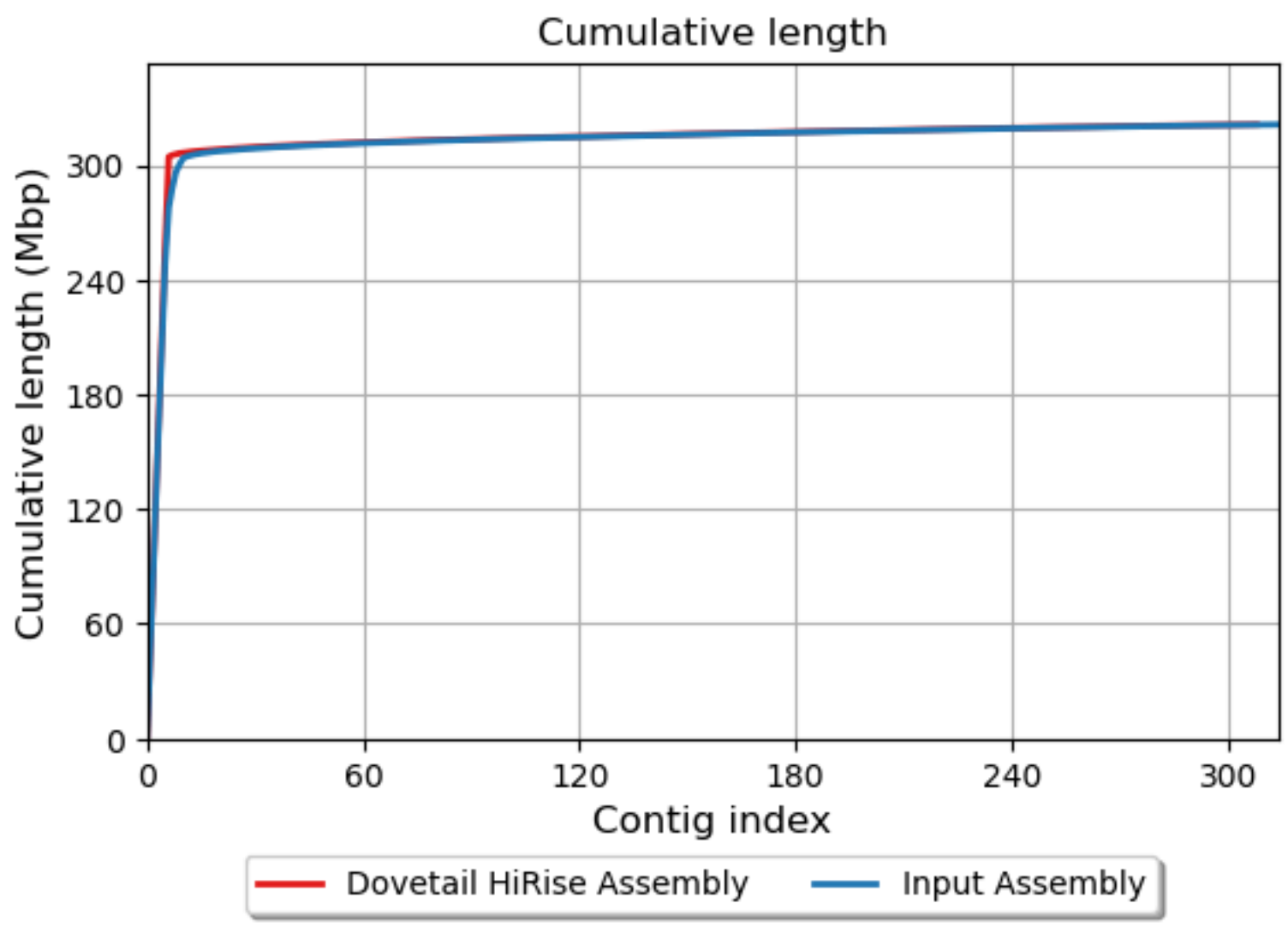

Contiguity Metrics

	Input Assembly	Dovetail HiRise Assembly
Largest scaffold	52,230,149	52,644,751
Number of scaffolds	314	308
Number of scaffolds > 1kbp	314	308
Number of gaps	0	6
Number of N's per 100 kbp	0.00	0.19

BUSCO

Assembly	Complete BUSCOs (C)	Complete and single-copy BUSCOs (S)	Complete and duplicated BUSCOs (D)	Fragmented BUSCOs (F)	Missing BUSCOs (M)	Total BUSCO groups searched
Input Assembly	250 (98.04%)	249 (97.65%)	1	2	3	255
Dovetail HiRise Assembly	250 (98.04%)	249 (97.65%)	1	2	3	255

- BUSCO version is: 4.0.5
- The lineage dataset is: eukaryota_odb10 (Creation date: 2020-09-10, number of species: 70, number of BUSCOs: 255)

Pair Size Distribution

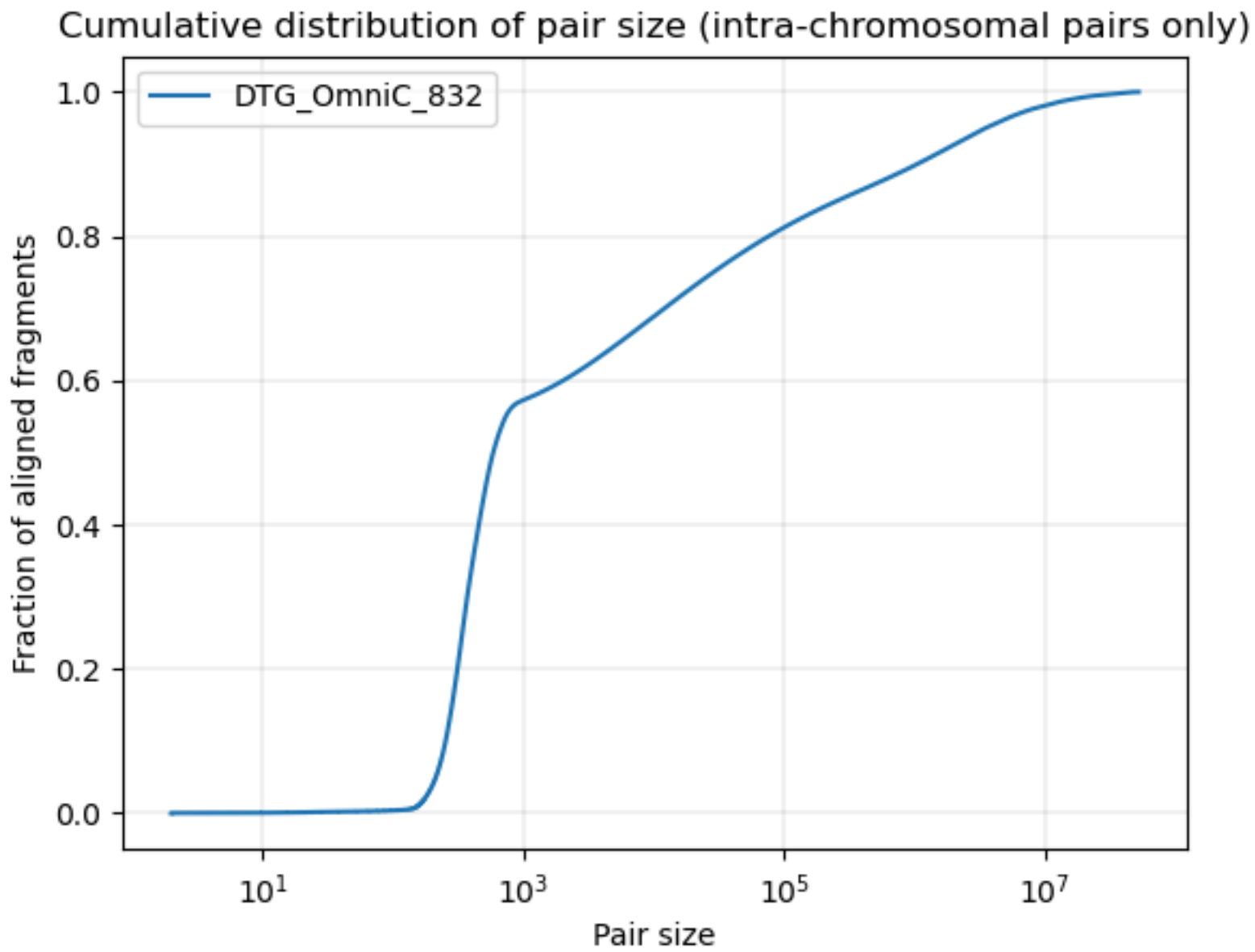

HiRise Scaffolding Information

Number of joins made by HiRise	6
Number of breaks made to input assembly by HiRise	0
Read-pairs	15,734,440

Materials and Methods

Dovetail Omni-C Library Preparation and Sequencing

For each Dovetail Omni-C library, chromatin was fixed in place with formaldehyde in the nucleus and then extracted. Fixed chromatin was digested with DNAse I, chromatin ends were repaired and ligated to a biotinylated bridge adapter followed by proximity ligation of adapter containing ends. After proximity ligation, crosslinks were reversed and the DNA purified. Purified DNA was treated to remove biotin that was not internal to ligated fragments. Sequencing libraries were generated using NEBNext Ultra enzymes and Illumina-compatible adapters. Biotin-containing fragments were isolated using streptavidin beads before PCR enrichment of each library. The library was sequenced on an Illumina HiSeqX platform to produce a approximately 30x sequence coverage. Then HiRise used MQ>50 reads for scaffolding (see "read-pair" above for figures).

Scaffolding the Assembly with HiRise

The input de novo assembly and Dovetail OmniC library reads were used as input data for HiRise, a software pipeline designed specifically for using proximity ligation data to scaffold genome assemblies (Putnam et al, 2016). Dovetail OmniC library sequences were aligned to the draft input assembly using bwa (https://github.com/lh3/bwa). The separations of Dovetail OmniC read pairs mapped within draft scaffolds were analyzed by HiRise to produce a likelihood model for genomic distance between read pairs, and the model was used to identify and break putative misjoins, to score prospective joins, and make joins above a threshold.

References

1. Putnam NH, O'Connell BL, Stites JC, Rice BJ, Blanchette M, Calef R, Troll CJ, Fields A, Hartley PD, Sugnet CW, Haussler D, Rokhsar DS, Green RE. Genome Research. 2016 Mar;26(3):342-50.
